# Quantitative Characterization of the T Cell Receptor Repertoire of Naïve and Memory Subsets Using an Integrated Experimental and Computational Pipeline Which Is Robust, Economical, and Versatile

**DOI:** 10.3389/fimmu.2017.01267

**Published:** 2017-10-12

**Authors:** Theres Oakes, James M. Heather, Katharine Best, Rachel Byng-Maddick, Connor Husovsky, Mazlina Ismail, Kroopa Joshi, Gavin Maxwell, Mahdad Noursadeghi, Natalie Riddell, Tabea Ruehl, Carolin T. Turner, Imran Uddin, Benny Chain

**Affiliations:** ^1^Division of Infection and Immunity, University College London, London, United Kingdom; ^2^UCL Cancer Institute, University College London, London, United Kingdom; ^3^Unilever Safety and Environmental Assurance Centre, Unilever, Sharnbrook, United Kingdom; ^4^School of Biosciences and Medicine, University of Surrey, Guildford, United Kingdom

**Keywords:** T cell receptor, repertoire analysis, naive T cells, memory T cells, unique molecular identifier

## Abstract

The T cell receptor (TCR) repertoire can provide a personalized biomarker for infectious and non-infectious diseases. We describe a protocol for amplifying, sequencing, and analyzing TCRs which is robust, sensitive, and versatile. The key experimental step is ligation of a single-stranded oligonucleotide to the 3′ end of the TCR cDNA. This allows amplification of all possible rearrangements using a single set of primers per locus. It also introduces a unique molecular identifier to label each starting cDNA molecule. This molecular identifier is used to correct for sequence errors and for effects of differential PCR amplification efficiency, thus producing more accurate measures of the true TCR frequency within the sample. This integrated experimental and computational pipeline is applied to the analysis of human memory and naive subpopulations, and results in consistent measures of diversity and inequality. After error correction, the distribution of TCR sequence abundance in all subpopulations followed a power law over a wide range of values. The power law exponent differed between naïve and memory populations, but was consistent between individuals. The integrated experimental and analysis pipeline we describe is appropriate to studies of T cell responses in a broad range of physiological and pathological contexts.

## Introduction

The adaptive immune system of jawed vertebrates uses imprecise somatic DNA recombination to generate a rich and diverse array of antigen specific receptors on B cells (BCR) and T cells (TCR). Although the mechanisms for the generation of diversity have been studied in great detail, the very diversity of the sequences coding for the receptors prevented a global analysis of the repertoire of B or T cell antigen receptors using conventional DNA sequencing techniques. The rapid advances in high-throughput DNA sequencing (HTS) over the past decades, and specifically the introduction of reliable massively parallel technologies [reviewed in Ref. (1)] have opened the way for increasingly robust and extensive BCR and TCR repertoire studies. Repertoire analysis provides a powerful tool for the study of both fundamental and translational immunology (2–5). Nevertheless, repertoire analysis provides many experimental and computational challenges, for which various solutions have been proposed (4, 6–8). Logistic considerations such as cost, ease of use, robustness, and versatility, as well as more scientific issues such as accuracy and coverage, may contribute to which solution is optimal for different laboratories.

In this paper, we present an integrated experimental and computational pipeline for TCR repertoire analysis and use it to provide a quantitative description of the repertoire in different sorted populations of human memory and naïve T cells. We do not present here a side-by-side comparison of this pipeline with other available techniques, although a study is currently in progress. Rather, we present a protocol which we and others have found useful with the hope that others in the community may also find it useful. All steps of the protocol are fully open source and as such can be used as they are, or developed further. We provide a series of quality control (QC) procedures which can be used to check the protocol at each step. The protocol is economical, robust, and has proved adaptable to different input sources of RNA, including whole blood, isolated peripheral blood (PB) lymphocytes, and solid tissue samples (including skin and lymph node biopsies, and tumor resections). The defining feature of the protocol is that it uses single-stranded cDNA ligation mediated by RNA T4 ligase (9), which we demonstrate to have a high efficiency, in order to incorporate unique molecular identifiers (UMIs) (10–12). UMIs can be used both for sequence error correction and to mitigate for inherent PCR heterogeneity (13). This allows quantitative estimates of TCR gene abundance. A suite of software tools (in Python) are provided for demultiplexing the output of Illumina sequencers, V and J gene assignment, error correction, and CDR3 extraction. These tools can be run individually or as a continuous pipeline.

The combined pipeline has been used here to analyze sorted naïve, central memory, and effector memory human PB T cell populations. Contrary to some previous reports, we find that after error correction, the naïve CD4 and CD8 population are made up predominantly of rare clones, of which over 95% are found only once in the sample analyzed. After error correction, the population structure of both memory and naïve T cells can be well-described by a power law distribution. The power law exponent of naïve populations is >4, while that of effector memory populations is around 2. Interestingly, CD4 central memory populations fall on a distribution of intermediate slope, while the distribution of CD8 central memory is similar to that of CD8 effectors. The corrected distributions also give robust and reproducible estimates for diversity indices. Finally, we demonstrate the presence of rare CMV-specific CDR3 sequences in the repertoire of two CMV positive individuals. Although a number of public or semi-public CMV CDR3s can be observed in the repertoires, the largest expansions of CMV-specific CDR3s only occurred in one individual. The study demonstrates the potential of this method to generate economic, robust, and quantitative TCR sequence data without recourse to proprietary technologies.

## Methods

### Sample Collection

This study was carried out in accordance with the recommendations of the UK Research Ethics Committee with written informed consent from all subjects. All subjects gave written informed consent in accordance with the Declaration of Helsinki. The protocol was approved by University College London Hospital Ethics Committee 06/Q0502/92.

### PB Fractionation

Peripheral blood mononuclear cells (PBMC) of three donors were sorted into memory and naïve CD4+ and CD8+ populations. Briefly, 120 ml of blood was collected from consenting healthy volunteers and PBMC were isolated by density-gradient centrifugation using Ficoll-Paque PLUS (GE Healthcare Lifescience). CD4 positive selection using Miltenyi beads was followed by CD8 positive selection of the CD4 negative fraction following the manufacturers’ instructions. CD4+ and CD8+ PBMC were stained with the following antibodies (all BD biosciences): CD3-PE-Cy7, CD4-V450, CD8-AF700, CD45RA-PE-CF594, and CD27-APC, as well as the fixable nearIR live/dead cell stain (Invitrogen). Cells were gated on lymphocytes, singlets, live cells, CD3+ and either CD4+ or CD8+ and then sorted into naïve T cells (CD45RA high, CD27+), central memory (CM, CD45RA−, CD27+), effector memory (EM, CD45RA−, CD27−) and effector memory RA-expressing revertants (EMRA, CD45RA+, CD27−) on an Aria II (BD). Sorted cell populations were centrifuged, the supernatant was removed and the cells were lysed and RNA stabilized in RLT buffer (Qiagen) following the manufacturer’s instructions. RNA was isolated using an RNeasy extraction kit following the manufacturer’s instructions (Qiagen) and stored at −80°C. Details of the samples collected and the RNA yield are shown in Table S1 in Supplementary Material.

The CMV status of donors was obtained by the overnight stimulation of fresh PBMCs with CMV viral lysate and identification of IFNγ production by CD4+ T cells as described previously (14). There was total concordance between IFNγ responses and seropositivity from IgG serology obtained from the diagnostic laboratory of University College London Hospital.

### KT2 T Cell Clone

The KT2 T cell clone which is specific for tetanus toxoid was a kind gift of Dr. Antonio Lanzavecchia and was cultured as described (15). The alpha and beta chains were amplified using steps 1–6 of the pipeline described below and cloned into pGEM T Easy Vector. The insert, which contains the full-length alpha and beta transcripts running from the alpha and beta RC1 primers until the 5′ end ligated to the SP2 ligation oligonucleotide, was cut out using the NotI sites on each side of the cloning site. The insert fragment was purified, quantified by spectrometry and then a series of dilutions were made up for use as standards for PCR1 and PCR2. These plasmids are freely available from the authors, and the full sequences and restriction maps are available at https://github.com/innate2adaptive/KT2-TCR-sequences.

### Skin Biopsies

Skin biopsies from tuberculin skin tests (TST) or saline-injected controls were collected and processed for RNA extraction as described in Ref. (16, 17).

### Unfractionated Whole Blood Samples

For whole blood analyses, 2.5 ml of PB from consenting adult healthy volunteers was drawn into Tempus tubes, and RNA extracted as per the manufacturer’s instructions (Thermofisher scientific).

### TCR Library Preparation

**Figure 1:**
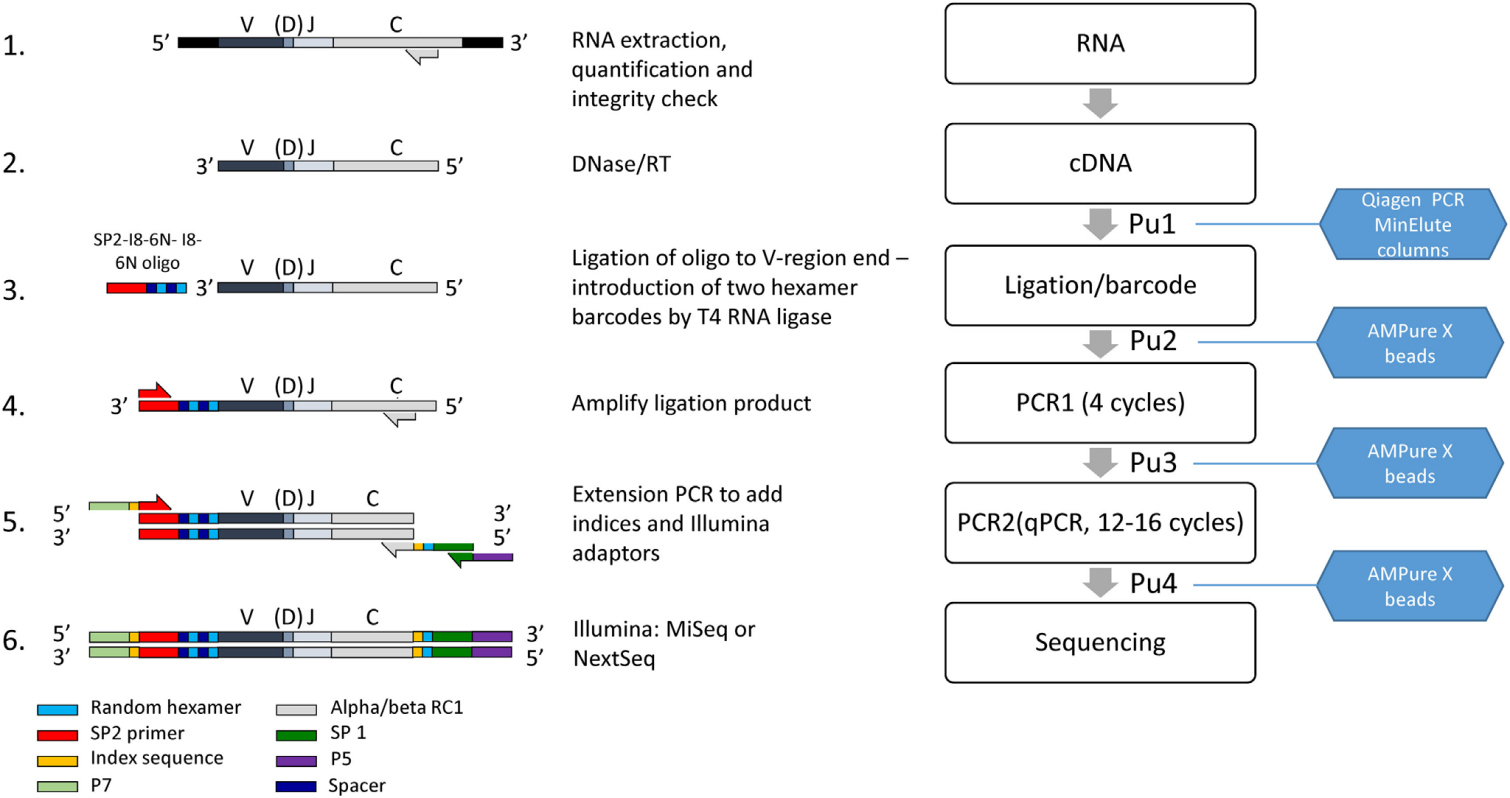
The laboratory pipeline for T cell receptor (TCR) sequencing. 1. RNA is extracted from cells or tissues, using standard protocols, quantified and checked for integrity. 2. Residual DNA is removed by DNAse treatment, and TCR RNA is then reverse transcribed into cDNA using primers close to the 5′ end of the constant region. 3. An oligonucleotide containing the Illumina SP2 sequencing primer, and a unique molecular identifier (UMI) consisting of two sets of six random nucleotides separated by spacers as shown, is ligated to the 3′ end of the cDNA using T4 RNA Ligase I. 4. The ligated product is amplified by four rounds of PCR, using nested primers in the alpha and beta C region in combination with the SP2 primer. 5. The product is further amplified and extended to incorporate the SP1 sequencing primer, indices for multiplexing and adaptors as shown. The final purified product is mixed with other indexed samples to give the final library, analyzed by capillary electrophoresis and sequenced on an Illumina MiSeq or Nextseq. PuX indicate the various purification steps.

A diagrammatic outline of the pipeline is shown in Figure 1, and detailed descriptions of each step are outlined below.

*Step 1* (Figure 1, line 1). Extract high-quality total RNA from a population of T cells, using standard silica membrane columns (typically RNeasy Mini or Micro kits, Qiagen). RNA should then be quantified fluorometrically (e.g., using the Qubit with RNA BR reagents, ThermoFisher Scientific) and integrity assessed using a Bioanalyzer or Tapestation (Agilent Genomics) or gel electrophoresis.

*Step 2* (Figure 1, line 2). DNase treatment. Mix 8 µl RNA, 1 µl RQ1 DNase (Promega, #M6101), and 1 µl RQ1 10× Buffer for 30 min at 37°C. Add 1 µl RQ1 DNase stop buffer and incubate at 65°C for 10 min in order to inactivate all DNase activity. The RNA should be diluted as necessary in RNase-free water to give a final volume of 8 µl, aiming for a final total amount of 1–1000 ng. If the RNA has been treated with DNase separately, this step is omitted, and the protocol starts at step 3 with 11 µl total RNA.

*Step 3* (Figure 1, line 2). Reverse transcription (RT). Add RNase-free water (4 µl), dNTPs (1.5 µl of a stock solution at a concentration of 10 mM for each dNTP, Promega) and RT primers alpha RC2 and beta RC2 (1.5 µl of each 10 µM stock, Table S2 in Supplementary Material) to the 11 µl of DNase treated RNA from step 2. The primer mix is denatured by heating to 65°C for 5 min, and then immediately and rapidly cooled on ice. Add Superscript (SS) reverse transcriptase (Invitrogen ThermoFisher) (1.5 µl), RNasin (1.5 µl, Promega #N2111), dithiothreitol (1.5 µl, 0.1 M), and Superscript FS buffer (6 µl of 5× stock) and incubate at 55°C for 30 min, and then 70°C for a further 15 min. Most of the experiments shown in this paper were carried out with SS III enzyme. However, the samples shown in Figure 4C were reverse transcribed with SSIV, according to the manufacturer’s protocol (10 min at 55°C, then 10 min at 80°C).

It is often useful to be able to quantify the amount of TCR mRNA in the sample. The alpha and beta RC2 primers are close to the start of the J region, and the resulting fragment of constant region cDNA produced is too short for quantitative real-time PCR (qPCR). However, alternative constant region primers (hTRAC_Q_R and hTRBC_Q_R, Table S2 in Supplementary Material) can be used for the RT reaction, and the resulting cDNA can be quantified using the additional hTRAC and hTRBC forward and probe oligonucleotides (Table S2 in Supplementary Material). These primer sequences were obtained from Ref. (18).

*Step 4* (Figure 1 Pu1) Purification 1. The RT mixture (30 µl total volume) is diluted with 150 µl of PB buffer from the MinElute PCR purification kit (Qiagen, # 28004) and the cDNA purified following the manufacturer’s instructions. Purified DNA is eluted in 10.5 µl water, which should be allowed to stand for 1 min prior to centrifugation as detailed in the Qiagen instructions.

*Step 5* (Figure 1 Line 3). Ligation. It is important that eluted cDNA samples be used for ligation within 24 h of purification, since substantial losses have been observed if samples are frozen and then thawed. The ligation mixture contains T4 RNA ligase 1 (2 µl, NEB, #MO204), T4 RNA ligase buffer (3 µl), bovine serum albumin (BSA)/hexamine cobalt chloride (HCC) mixture (1 mg/ml BSA; 10 mM HCC, 3 µl), 10 mM ATP (1 µl), ligation oligonucleotide (1 µl, 10 µM stock), PEG8000 (10 µl, 50% stock solution, supplied with T4 RNA ligase 1), and 10 µl of purified cDNA from *step 3*. The ligation mixture is very viscous due to the high concentration of PEG8000, and requires slow and thorough mixing. The ligation mixture is incubated at 16°C for 20–24 h, and then stored at 4°C until the purification.

**Figure 2:**
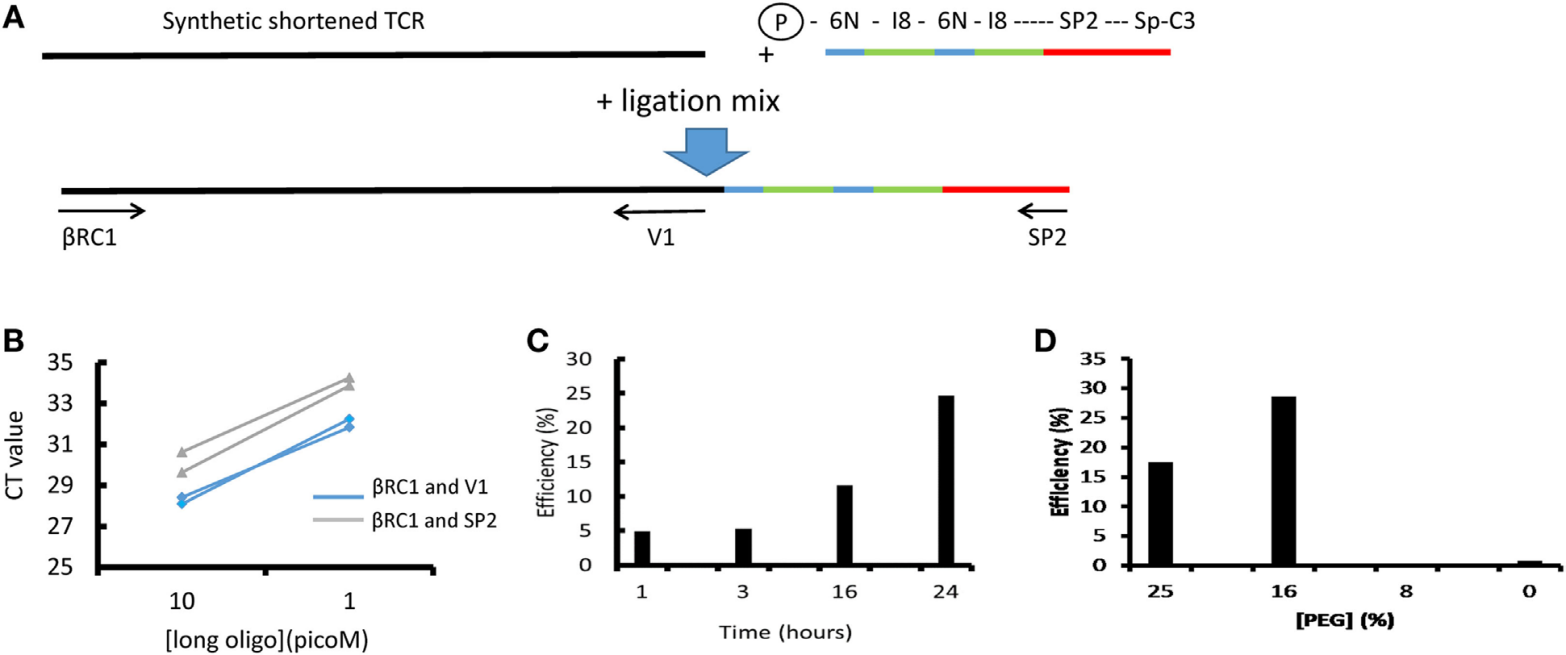
The efficiency of T4 RNA ligation. **(A)** Schematic of ligation assay. A long (250 bp) oligonucleotide (LO) coding for a short version of the Jurkat TCRβ gene was ligated to the unique molecular identifier (UMI)-SP2 oligonucleotide as described in the Section “Methods.” After ligation and bead purification the total amount of LO and the amount of ligated LO were measured separately by SYBR Green qPCR using βRC1 and V1 or βRC1 and SP2 primers, respectively. The efficiency is calculated as the ratio of ligated product, relative to total LO in the reaction, as determined by the βRC1-SP2 and βRC1-V1 amplification qPCRs. **(B)** A representative result showing the CT values obtained at two concentrations of LO, in two repeat qPCRs (blue: ligated product; green: total LO). **(C)** The efficiency of the ligation as a function of ligation time. **(D)** The efficiency of the ligation as a function of final polyethylene glycol (PEG) concentration in ligation mix.

The standard ligation conditions are based on Ref. (19, 20). The efficiency and sensitivity to various parameters has been explored using a quantitative ligation assay as described below and Figure 2. The ligation oligonucleotide sequence is shown diagrammatically in Figure 2A and the full sequence is given in Table S2 in Supplementary Material. It contains the Illumina SP2 primer region (which acts to prime the reverse sequencing read 2), followed by an 8 base spacer, and a 12 base UMI. The 12 nucleotide UMI consists of two stretches of six random nucleotides, separated by an eight-base spacer that acts to prevent hairpin formation within the UMI. The ligation oligonucleotide is phosphorylated at the 5′ terminus, which is a requirement of T4 RNA ligase 1, and blocked at the 3′ terminus with a Spacer C3 moiety to prevent oligonucleotide concatemerization (Figure 2A).

*Step 6* (Figure 1 Pu2). Purification 2. It is critical to remove as much of the ligation oligonucleotide as possible prior to subsequent amplification steps. This purification, and all subsequent purification steps are carried out using Agencourt AMPure XP magnetic beads (BeckmanCoulter A63880) according to the manufacturer’s instructions. Since there is PEG in the ligation mixture, and there is also PEG in the AMPure magnetic bead purification solution, the relative volumes of beads and DNA solution have been altered as described. The beads must be allowed to warm up to room temperature for at least 30 min before use.

The ligation mix (30 µl) is diluted by addition of 70 µl RNase-free water and mixing well. 50 µl AMPure beads are added to 100 µl of diluted ligation product, and mixed thoroughly by pipetting up and down 10 times or until homogeneous. The bead/DNA mixture is left to stand for 5 min at room temperature. The beads are collected by placing tubes (for small numbers of samples) or plates (for larger number of samples) on a magnetic stand for 2 min. The liquid above the beads is carefully aspirated and discarded. The beads are washed twice with 300 µl of fresh 80% ethanol (EtOH), air-dried for less than 5 min (typically 3–4) and the DNA is eluted in 33 µl molecular grade H_2_O. Quantitative measurements of cDNA concentrations before and after purification indicate the purification yield at this step is between 0.5 and 0.7.

*Step 7* (Figure 1 line 4). PCR1. If possible PCR1 should be set up on the same day as the purification of the ligation mixture. 31 µl of the purified ligation mixture is mixed with dNTPs (1 µl, 10 mM stock), primers (2.5 µl, 10 µM stock), Phusion High Fidelity proofreading DNA polymerase (0.5 µl Thermofisher, #F530), and Phusion HF buffer (10 µl, 5× stock). The PCR mixture (final volume 50 µl) contains three primers (see Table S2 in Supplementary Material), SP2—which anneals to the corresponding sequence introduced by the ligation oligonucleotide plus alpha RC1 and beta RC1, which hybridize to the constant region of the alpha and beta genes, respectively. Note that these oligonucleotides map 5′ to the constant region primers used for the RT, thus acting as nested primers in relation to the RT step. This proved to be an essential feature of the protocol, significantly increasing the specificity of the amplification step. Note further that the beta RC1 primer is an equimolar mixture of two oligonucleotides, beta RC1.1 and beta RC1.2. This provides coverage of both the TRBC1 and TRBC2 beta chain constant region alleles, which differ by 2 bp in this region (21), while avoiding the additional sequences and unknown relative concentrations that would be produced by ordering an oligonucleotide with a degenerate sequence.

PCR cycling parameters are heated lid (typically 105°C), 98°C 3 min denaturation step, followed by 4 cycles of 98°C, 15 s denaturation, 69°C, 30 s annealing, and 72°C, 40 s extension; followed by 72°C, 5 min final extension. The samples can be stored at −20° after this step if required.

*Step 7a* (optional QA). In order to test whether the ligation step has been successful, a qualitative PCR can be performed, using 1 or 2 µl of purified ligation mix, and the same PCR conditions as above (make up to total volume of 50 µl with water). The PCR should be run for 30 cycles and should generate a band of about 550 bp.

*Step 8* (Figure 1 Pu3). Purification 3. Mix the whole of the PCR1 mixture (50 µl) with 40 µl AMPure XP beads (prewarmed and thoroughly resuspended). Proceed with bead purification protocol as for purification 2. Elute in 31 µl water.

*Step 9* (Figure 1 line 5). PCR2. An extension PCR that incorporates the Illumina sequencing adaptors P5 and P7 and the sequencing primer SP1 (sequencing read 1) sequence. In addition, it incorporates two indexing sequences that allow multiplexing of many different samples on the same sequencing run. The index 5′ to SP2 is read by Illumina sequencers during an additional short sequencing step between sequencing reads 1 and reads 2 (index read 1). An additional index is used in this protocol, inserted immediately 3′ to the constant region, which is read at the beginning of sequencing read 1. One representative index sequence we have used at each index position within the amplicon is shown in Table S2 in Supplementary Material, but other indices can be used as required. The Illumina sequencing technology requires diversity in the first few bases in order to obtain good cluster identification. An additional 6 random bp are, therefore, incorporated immediately 5′ to SP1 as shown.

T cell receptor alpha and beta chains are processed separately from this stage. 12.4 µl of purified PCR1 ligation mixture is mixed with dNTPs (0.62 µl, 10 mM stock), ROX reference dye (0.5 µl, Thermofisher # 12223012), SYBR Green solution (2.5 µl), primers SP1-P7, P7-I*X*-SP2 (1.25 µl, 10 µM stock, I*X* refers to an index number), primer SP1-6N-I*X*-aRC1 or SP1-6N-I*X*-bRC1 (1.25 µl, 1 µM stock, Table S2 in Supplementary Material, *X* refers to another index number), Phusion High Fidelity proofreading DNA polymerase (0.25 µl Thermofisher, #F530), and Phusion HF buffer (5 µl). Total volume 25 µl.

PCR cycling parameters are heated lid, 98°C 3 min denaturation step, followed by cycles of 98°C, 20 s denaturation, 69°C, 30 s annealing, and 72°C, 40 s extension. The reaction is stopped when the fluorescence signal reaches a prespecified threshold (see below). The samples can be stored at −20° after this step if required.

The PCR mixture (final volume 25 µl) contains three primers (see Table S2 in Supplementary Material; Figure 1). The two outer primers SP1-P5 and P7-I*X*-SP2 are used at a stock concentration of 10 µM, while the inner SP1-6N-I*X*-aRC1 or SP1-6N-I*X*-bRC1 is present at 10-fold lower concentration to favor the outer reaction.

The SYBR Green stock solution (SYBR Green I Nucleic Acid Gel Stain 10,000× concentrate in DMSO, Invitrogen/ThermoFisher) is first diluted 1:100 in DMSO. This solution is stored is at −20°C, and then an aliquot is further diluted 1:50 in water immediately before addition to PCR mixture.

A DNA fragment standard (typically 12.5 pg) of a DNA fragment consisting of a ligated KT2 TCR sequence from the SP2 to the RC1 sequence (see above and Figure 1) is amplified in parallel to new samples in PCR2 as part of QC. This sequence is produced through the extended amplification of PCR1 using KT2 mRNA as a target.

PCR2 is typically carried out as a SYBR Green qPCR, allowing the progress of the reaction to be observed in real time, and the cycler stopped when the signal reaches a predetermined threshold (see example of run in Figure S1 in Supplementary Material; the threshold is selected to lie within the logarithmic expansion phase of the amplification). In this way, over amplification is prevented. In cases with sufficient T cell numbers/RNA concentration, this standard PCR2 produces enough library DNA for sequencing, in which case these preparations can proceed directly to step 10.

In samples with low T cell/TCR mRNA input, PCR2 can be carried out as a conventional end-point reaction (typically for 6 cycles). This PCR2 product is then purified using purification 3 protocol (PU3, step 8), and further amplified in a qPCR using P5s and P7 primers. For PCR3 we use 13.6 µl sample + 11.4 µl mastermix, containing buffer, enzyme, dNTP, Rox, and Cyber green as described for PCR2, plus 1.25 µl P5s primer (10 µM stock) and 1.25 µl P7 primer (10 µM stock) using the reaction conditions described above.

*Step 10* (Figure 1 Pu4). Purification 4. Mix the whole of the PCR2 mixture (25 µl) with 20 µl AMPure XP beads (rewarmed and thoroughly resuspended). Proceed with bead purification protocol as for purification 2. Elute in 31 µl water.

*Step 11* (Figure 1, line 6). Sequencing. The concentration of each DNA sample after amplification is quantified by spectroscopy (dsDNA high-sensitivity Qubit kit, ThermoFisher Scientific). Samples are also analyzed by micro-electrophoresis using a high-sensitivity D1000 TapeStation screen tape (Agilent). Typical profiles are shown in Figure S1 in Supplementary Material. Successful library preparation yields a major peak of approximately 650 bp as predicted from the combined size of the recombined TCR variable gene, together with the short sequence of constant region and various adaptors/indices. The peak molecular size as determined on the Tape station is used to convert the concentration from ng/μl to nM. The final libraries are prepared by mixing samples with different indices, such that each sample is present at 4 nM in the final library. Typically, 12 samples are run in parallel, typically yielding 1–2 million reads per sample when using a v2 kit (2 × 251 paired end) on an Illumina MiSeq. However, greater depth can be achieved by running fewer samples per flow cell where needed. The number of UMIs in a sample increases with read depth (Figure S2 in Supplementary Material) although as shown the number begins to saturate around 1–2 million reads for PB repertoires. Where necessary, samples can be further concentrated either by adding additional PCR cycles (amplifying with P5 and P7 primers after PCR2), or by drying DNA down to a smaller volume. The final 4 nM library is then prepared for sequencing using the standard Illumina MiSeq protocol. 5–7% PhiX DNA is added to the library to increase diversity and improve cluster recognition.

### TCR Analysis Protocol

**Figure 3:**
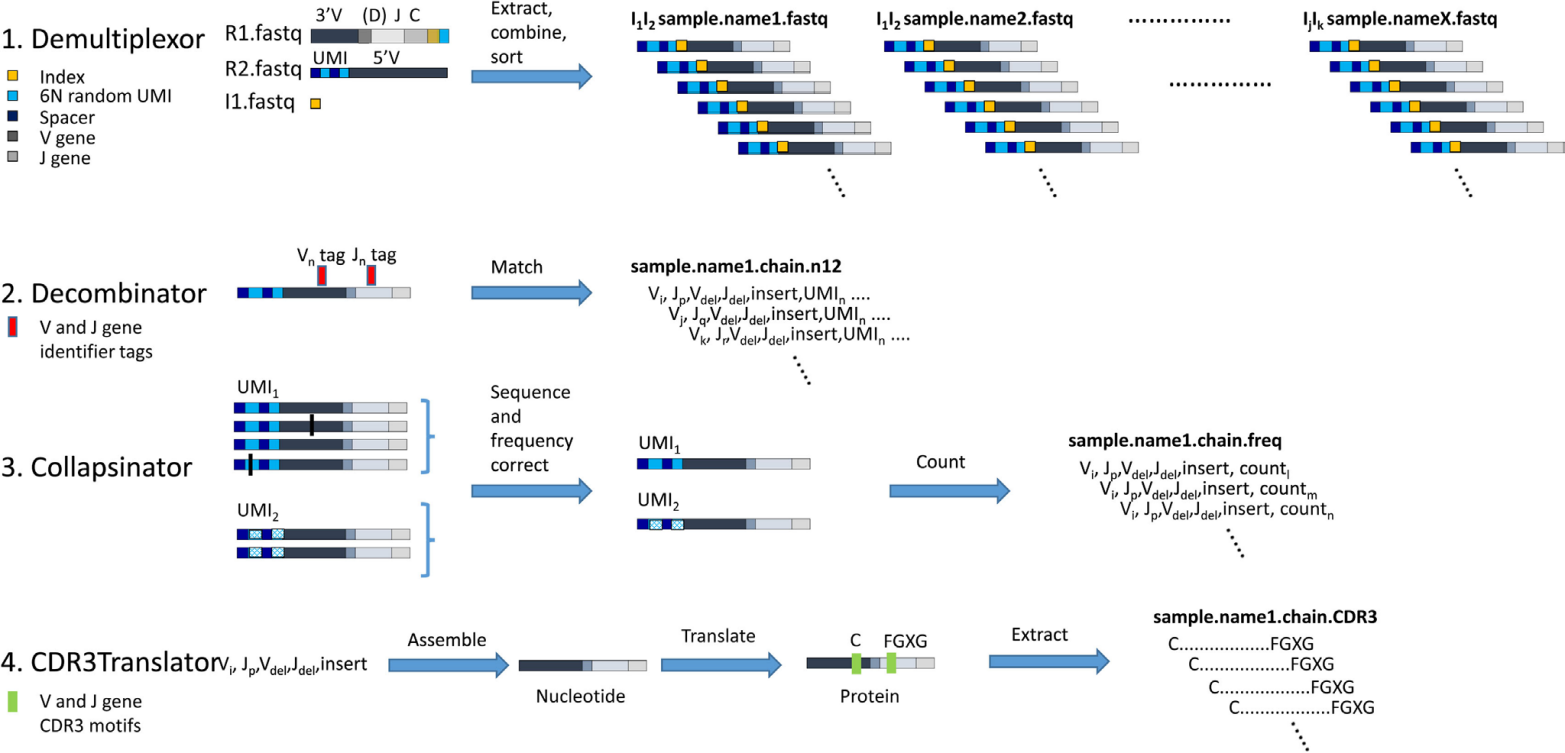
The computational pipeline for T cell receptor (TCR) analysis. A diagrammatic representation of each of the four stages of analysis, each performed by an independent software module as shown. 1. Demultiplexor takes raw FASTQ files generated by the Illumina sequencer, extracts the unique molecular identifier (UMI) sequence from read 2 and combines it with the TCR sequence from read 1, and then demultiplexes the reads into sets of FASTQ files each containing data from only one indexed sample. 2. Decombinator takes individual FASTQ TCR reads and identifies the relevant V and J genes, and the V–J junctions (which encompass the D genes for beta chains, but do not explicitly identify them). Each TCR is assigned a five-part identifier, V_i_, a number denoting the V region (corresponding V region names according to IMGT nomenclature are given in Table S3 in Supplementary Material); J_p_, a number denoting the J region (corresponding V region names according to IMGT nomenclature are given in Table S3 in Supplementary Material); V_del_, the number of nucleotides deleted from the 3′ end of the genomic V gene sequence; J_del_, the number of nucleotides deleted from the 5′ end of the genomic J gene sequence; and insert, the nucleotide sequence between the end of the V and the beginning of the J (which includes any remaining D region nucleotides for beta and delta chains). These five identifiers together uniquely identify the recombined TCR nucleotide sequence. The unique UMI_n_ incorporated into each TCR read is appended to each TCR identified. 3. Collapsinator takes Decombinator output and uses the UMIs to correct both for sequencing errors and for frequency errors that arise from PCR heterogeneity. 4. CDR3Translator converts the Decombinator five-part identifier into nucleotide sequence, translates this sequence into amino acid sequence, and then extracts the CDR3 region based on their C-terminal and N-terminal motifs.

A diagrammatic outline of the analysis pipeline is shown in Figure 3. A detailed description of each step is outlined below:

The analysis of the FASTQ files is carried out using a suite of Python scripts available at https://github.com/innate2adaptive/Decombinator. The repository also contains help files and test data. The core TCR assignment is based on a modified version of the original Decombinator software (22), which uses a tag-based recognition method based on the Aho-Corrasick algorithm (23). The processing is broken down into four steps, which can be run independently (with separate scripts) or combined using a Makefile.^1^

1. *Demultiplexor.py* (Figure 3, top line). Illumina machines produce 3 read files from the libraries generated as described above: Read 1 (R1) contains the V(D)J sequence and a demultiplexing index (the SP1 index, Figure 1); Read 2 R2 contains the UMI sequences and reads into the 5′ UTR; Index 1 (I1) contains the second demultiplexing index (the typical Illumina index read, giving the SP2 index). The three files are converted to FASTQ format using the Illumina bcl2fastq conversion software.^2^ Note that in order to produce single R1/R2/I1 files for the entire runs, the run must either be performed without providing demultiplexing indexes or the reads from all samples must be pooled afterward. Production of separate I1 index files may also not be set as a default option on most users’ sequencing machines, which may require alteration of configuration files (which should be done by an experienced Illumina user).

The Demultiplexing script extracts the relevant sequences from each sequence and combines them into a single sequence, containing the first 30 bp of read 2 (covering the random barcode and spacer nucleotides), followed by two sets of hexamers (Illumina index read and index from R1), and the whole of R1 (which contains the end of the constant region and the majority of the variable region—see Figure 1). The script then searches for combinations of the indices specified by the user and separates out the reads into a set of separate FASTQ files, named according to corresponding sample identifiers supplied by the user. The output is, therefore, a set of FASTQ files, each corresponding to a distinct biological sample (optionally also a distinct TCR chain), plus a log file which summarizes the output.

The script requires the location of the three original FASTQ files R1, R2, and I1; and a CSV file containing the sample names and corresponding index sequence numbers as defined in Table S2 in Supplementary Material. The output is a series of FASTQ files, each containing a set of UMI and associated TCR sequences from a single biological sample. The file names incorporate the operator-determined sample names. These names will be carried downstream throughout the analysis, and so should be chosen with care. Including the chain (e.g., “alpha”) in the file name will allow auto-detection in subsequent scripts (if only one chain is used per file).

Further details about the data input and setting additional optional flags can be found as annotation inside the Demultiplexor script and in the accompanying README file.

2. *Decombinator.py* (Figure 3, line 2). This module is an improved version of the original Decombinator algorithm which is described in detail in Ref. (22). The algorithm uses a fast, key word-based algorithm to search FASTQ reads (e.g., produced through Demultiplexor.py) for rearranged TCR chains, assign V and J genes, and determine the junctional deletions/additions relative to the genomic gene sequence. Assignment is done by matching to V and J gene specific tags, allowing a single base pair mismatch as described in detail in the original paper.

Decombinator can currently analyze both human and mouse TCRs, analyzing both alpha/beta and gamma/delta chain rearrangements.

The input (provided *via* command line arguments) should include FASTQ files produced by Demultiplexor.py (unzipped or gzipped). Data produced from other sources can also be analyzed, but users will need to consult the documentation to correctly set the non-default settings. The TCR chain locus to look for can be explicitly specified using the -c flag and users can specify chain identifiers from a/b/g/d/or alpha/beta/gamma/delta or/TRA/TRB/TRG/TRD/or TCRA/TCRB/TCRG/TCRD. If no chain is provided (or if users wish to minimize input arguments), the script can infer the chain from the FASTQ filename, e.g., “alpha_sample.fq” would be analyzed for alpha chain recombinations.

There are several other optional input flags which control the output and analysis and can be found within the Decombinator.py script or can be accessed by viewing the help data, by running Decombintator.py –h.

The output is a CSV file, with a name based on the file name allocated in Demultiplexor, and the default extension.n12 (referring to the 12 random nucleotides of the UMI). Each line of output corresponds to a distinct TCR sequence and contains the following 10 fields:

1. A number denoting the V region (corresponding V region names according to IMGT nomenclature are given in Table S3 in Supplementary Material).
2. A number denoting the J region (corresponding V region names according to IMGT nomenclature are given in Table S3 in Supplementary Material).
3. The number of nucleotides deleted from the 3′ end of the genomic V gene sequence.
4. The number of nucleotides deleted from the 5′ end of the genomic J gene sequence.
5. The nucleotide sequence between the end of the V and the beginning of the J (which includes any remaining D region nucleotides for beta and delta chains).
6. The Illumina FASTQ line identifier, so as to be able to link the output back to the original raw sequence data if necessary.
7. The complete nucleotide sequence between the V gene and J gene tags used for V and J identification (the “intertag sequence”), which is used for error correction.
8. The corresponding quality of each base in the intertag sequence as determined by the Illumina sequence (as Phred score).
9. The first 30 bases of the R2 read, containing the spacer and UMI sequences from the ligation oligonucleotide as shown in Figure 2.
10. The corresponding read quality of the UMI sequence.

The script also outputs a log file, which summarizes the statistics for the sample, and includes a lower bound estimate of error rate per base pair around the relevant portion of the TCR molecule/read, obtained by counting the number of single base pair mismatches between tag and read sequences.

*Collapsinator.py* (Figure 3, third line). This script takes the output files of Decombinator and performs qualitative and quantitative error correction mitigating for both sequencing error and PCR amplification heterogeneity.

1. The output from Decombinator is grouped according to UMI, and within each UMI group the TCR sequences are error-corrected as follows:
  - The TCR sequences are ordered in descending abundance order, and the most common is considered first (or if there are multiple sequences of equally large abundance, one is chosen arbitrarily). All other reads within the UMI group are considered in turn, and those with an intertag sequence that differs from the most common by less than a pre-assigned proportion of their bases are assumed to have derived from the same single TCR mRNA molecule by a process of PCR or sequencing error. As such these reads are incorporated into the most common TCR sequence within the UMI and do not represent different TCR rearrangements in the sample. We assume that the most frequent variant, rather than any of the minor variants, represents the true TCR sequence since most errors are likely to occur later during the PCR process (when more molecules are present) or during the sequencing and, thus, will appear as in the sequencing output as minor variants, see Ref. (24). Any sequences that were not incorporated into the first common TCR get set aside for subsequent iterations.
  - The process described in (a) is repeated on the remaining TCR sequences within the UMI, taking the remaining next most abundant TCR as the reference sequence, proceeding iteratively until all sequences within the UMI have been considered.
2. Next, where the same (or similar, to a preset number of mismatches) TCR is associated with multiple UMIs, this set of UMIs is considered to see whether they likely derived from the same labeled TCR cDNA molecule by sequence/PCR error. Where UMIs differ in only a preset threshold of bases, they are assumed to represent the same initial TCR molecule and are clustered together.

This process provides, for each UMI, a set of TCR sequences that have likely derived from separate TCR cDNA (and, hence, separate mRNA) molecules. In practice, because of the diversity of UMIs, UMIs associated with more than one distinct TCR are rare.

This process provides UMI–TCR pairs that are believed to represent sequence output from a single initial TCR molecule. The number of different UMIs paired with a single TCR sequence provides the “clone size” of that TCR, giving a corrected estimate of the actual abundance of that molecule in the original ligation reaction. In addition, the number of Decombinator output lines that have been incorporated into each UMI–TCR pair provides a measure of the PCR amplification that the experimental pipeline has applied to the initial single TCR molecule. The number of sequences incorporated into each UMI–TCR pair is observed to be very heterogeneous (see Figure S3 in Supplementary Material), illustrating the considerable amount of PCR heterogeneity as we described previously (13).

The output of this script is a csv file, with a name based on the file name allocated in Demultiplexor, and the extension.freq. Each line of the output file contains the first five fields of the Decombinator output (see above), which provide unique TCR identifiers for the error-corrected sequences, with a sixth field containing the number of times this TCR is found within the sample. The script also generates a log file which contains a summary of the file statistics.

*CDR3Translator.py* (Figure 3, fourth line). This script takes the output files of Collapsinator.py or Decombinator.py and determines the corresponding protein sequence of the CDR3 hypervariable loop. This is defined by convention as the region from the second conserved cysteine encoded in the 3′ of the V gene to the phenylalanine in the conserved “FGXG” motif in the J gene of a given rearrangement. Some non-canonical CDR3 motifs have also been reported. The algorithm also identifies “non-productive” TCR sequences, i.e., those that contain stop codons, are out-of-frame, or lack any CDR3 motifs (for a full discussion of what constitutes a functional or productive TCR see http://www.imgt.org/IMGTScientificChart/SequenceDescription/IMGTfunctionality.html).

The algorithm takes the unique 5-part identifier produced by Decombinator (fields 1–5 defined above) and converts these into a full DNA sequence, based on defined sets of sequences for each V and J regions. These can be hosted locally during analysis or be automatically downloaded from https://github.com/innate2adaptive/Decombinator-Tags-FASTAs (files ending with a.fasta extension). The full DNA sequence is then translated into protein sequence, and the CDR3 sequences extracted. The identification of CDR3 motifs uses an additional set of files which define the position and sequence of V and J terminal motifs for each known V and J gene, which can also be found at the same repository as above (files ending with a translate extension). These files allow non-canonical CDR3s to be included.

The output consists of either cdr3 files, which consist of the unique productively-rearranged CDR3s from the original file, with their frequency, or “.dcrcdr3” files, which contains the five-part Decombinator index before the CDR3 and its frequency; this option is provided as multiple TCR rearrangements can encode the same CDR3 sequence. The choice of which file format is used is decided through use of the “dcroutput” variable (*via* the “-do” flag). The script also outputs some simple statistics about the number of productive rearrangements, and the frequency of different genes.

Further details of all the software, the current annotated versions of the scripts and all necessary accessory files can be found at the Innate2Adaptive GitHub repository. Note that the “Decombining” and translation functionalities can also be called from other Python modules, allowing incorporation of V/J assignment and CDR3 extraction into more complex bespoke analysis procedures.

All the sequences analyzed in this manuscript have been submitted to the Short Read Archive^3^ as experiments SRP108840 (KT2 sequences); SRP109035 (naive/memory), and SRP108891 (Skin samples).

## Results

### Library Preparation and Sequence Analysis

The twin pipelines for library preparation and subsequent sequence analysis are summarized in Figures 1 and 3. The details are described in the methods section above. The key features are outlined below:

1. cDNA synthesis. The protocol uses RNA rather than DNA, since this allows straightforward introduction of UMIs. The use of RNA also increases the likely coverage of the repertoire since each cell contains several molecules of TCR messenger RNA. The method is potentially sensitive to changes in RNA message associated with T cell activation and differentiation. However, several studies have demonstrated that such changes are small and transitory and are, therefore, unlikely to have a major impact on repertoire (24–27). We used a constant region qPCR to measure the number of TCR molecules per T cell in six samples of PBMC from healthy volunteers (Figure S4 in Supplementary Material). There were 180 ± 50 molecules of TCR alpha cDNA (mean ± SD, *n* = 6) and 400 ± 90 molecules of TCR beta cDNA per T cell. The ratio of beta to alpha was 2.3 ± 0.5. This figure is broadly consistent with similar measurements of mouse TCR beta chain message (24, 28) levels, and is also consistent with single-cell studies showing that TCR message is highly expressed (29, 30). We have also seen similar differences in alpha and beta message abundance when analyzing TCRs in total RNAseq data (not shown). The implications of RNA abundance and repertoire coverage are discussed in more detail below.
2. The ligation of an oligonucleotide to the 3′ end of the cDNA permits introduction of UMIs before PCR amplification and also avoids the need for multiplex PCR and the biases this can generate. This greatly increases the flexibility of the protocol because T cells from different species or gamma/delta TCRs—or indeed any target transcript—can be easily processed simply by changing the constant region primers. Although T4 RNA ligase is often described as having low efficiency for single-stranded DNA, we were unable to discover any publications with direct measurements of this efficiency. We, therefore, developed an assay for ligation efficiency, using as substrate a long (250 bp) oligonucleotide which coded for a shortened TCR beta chain from the Jurkat cell line. As shown in Figure 2, this assay suggested that the T4 ligase had efficiencies >10% under optimum conditions. We confirmed this by measuring ligation efficiency using full-length cDNA extracted from Jurkat cells. Thus, at least one in 10 TCR cDNA molecules should be ligated after overnight ligation using this method.
3. PCR. We carried out the amplification protocol in two steps. All PCR steps were carried out using Phusion high fidelity proofreading polymerase. We observed that we could significantly increase efficiency by introducing a washing step after four cycles of PCR1. This was not due to inherent low efficiency of this PCR, because amplification of an appropriate standard (a previously cloned TCR) showed PCR efficiencies >1.9. We speculate that residual oligonucleotide from the ligation step may remain after the first bead washing step and interfere with the amplification. The second PCR was carried out using a qPCR SYBR Green protocol (Figure S1 in Supplementary Material), so that amplification could be observed in real time. In this way, the amplification could be stopped when the amount of product reaches a predetermined threshold, corresponding to a sufficient yield of DNA for sequencing. This avoids excessive amplification, which could bias against some rare TCR sequences in the starting pool and minimizes the burden of erroneous sequence production.
4. PCR2 introduces several features required for subsequent sequencing on the Illumina platform, including the first sequencing primer SP1, an Illumina index for sequence multiplexing and the two Illumina anchor sequences P5 and P7. A random hexamer is introduced immediately downstream of SP1 to increase diversity at the beginning of the first sequence run, which greatly improves cluster identification. A second multiplexing index sequence is also introduced immediately 3′ of the constant region. The option to introduce two independent indices is important when considering high-throughput experiments with many samples using the NextSeq or HiSeq machines.
5. The cost of the protocol is an important consideration when large numbers of samples are to be processed. The approximate costs of the various materials required for the protocol are summarized in Table 1 (exact costings will depend on local pricing agreements). The current total cost is in the order of £14 (approximately 16–18 Euros or US dollars) per sample, excluding sequencing. The most expensive component is the reverse transcriptase SuperScript III. We have not experimented with other reverse transcriptases but it is possible the total cost could be further reduced by using a different source of this enzyme.
6. The analysis pipeline is built around the Decombinator software that has been described previously (22). DecombinatorV3 is significantly faster than previous versions, and by default uses an extended set of V and J regions described at https://github.com/innate2adaptive/Decombinator-Tags-FASTAs. In addition to the 5 part identifier described previously (V gene, J gene, number of V deletions, number of J deletions, and the sequence between the end of the V and J genes), it retains the first 30 bps of read 2 which contain the UMI sequence for subsequent analysis.
7. Three additional new utilities are provided. Demultiplexor uses a combination of the Illumina and the additional index sequence (provided by user) described above to separate different samples which have been sequenced in the same run. Collapsinator implements a UMI-based error correction procedure that is described in more detail in the Section “Methods,” and outputs a set of unique 5 part identifiers for each sample, together with their abundance in the sample. Finally CDR3translator translates and extracts the CDR3 from each error-corrected TCR. Each module provides a summary of the output in comma-delimited format. Each module can be run independently, or the full pipeline can be run simultaneously using a Makefile.

**Table 1:** The list of reagents and costs per sample (in pounds sterling) for the TCR library preparation.

Reagent	UCL cost per sample
Ampure beads	0.87
Bovine serum albumin	0.00
Cyber green	0.02
DNa away	0.12
dNTPs	0.14
Ethanol	0.00
Ligation oligo (HPLC)	0.01
Lowbind tubes	0.36
Mini elute kit	1.22
oligos	0.09
Phusion polymerase	1.04
Pipette tips	0.97
Qubit reagents	0.72
Q-PCR stripes	0.21
Qubit tubes	0.19
RNA ligase reaction buffer (+PEG/ATP)	0.32
Rnase away	0.04
RNAse-free water	0.02
RNAsin	0.70
Rox	0.03
RQ1 DNase	0.04
Superscript III RT mix	4.26
T4 RNA ligase	0.06
Tapestation reagents	0.53
Tapestation ScreenTapes	1.55
TapeStation Tips	0.18
	13.70

Further details of individual reagents are given in the protocol as detailed in Section “Methods.”

### Pipeline Sensitivity and Versatility

**Figure 4:**
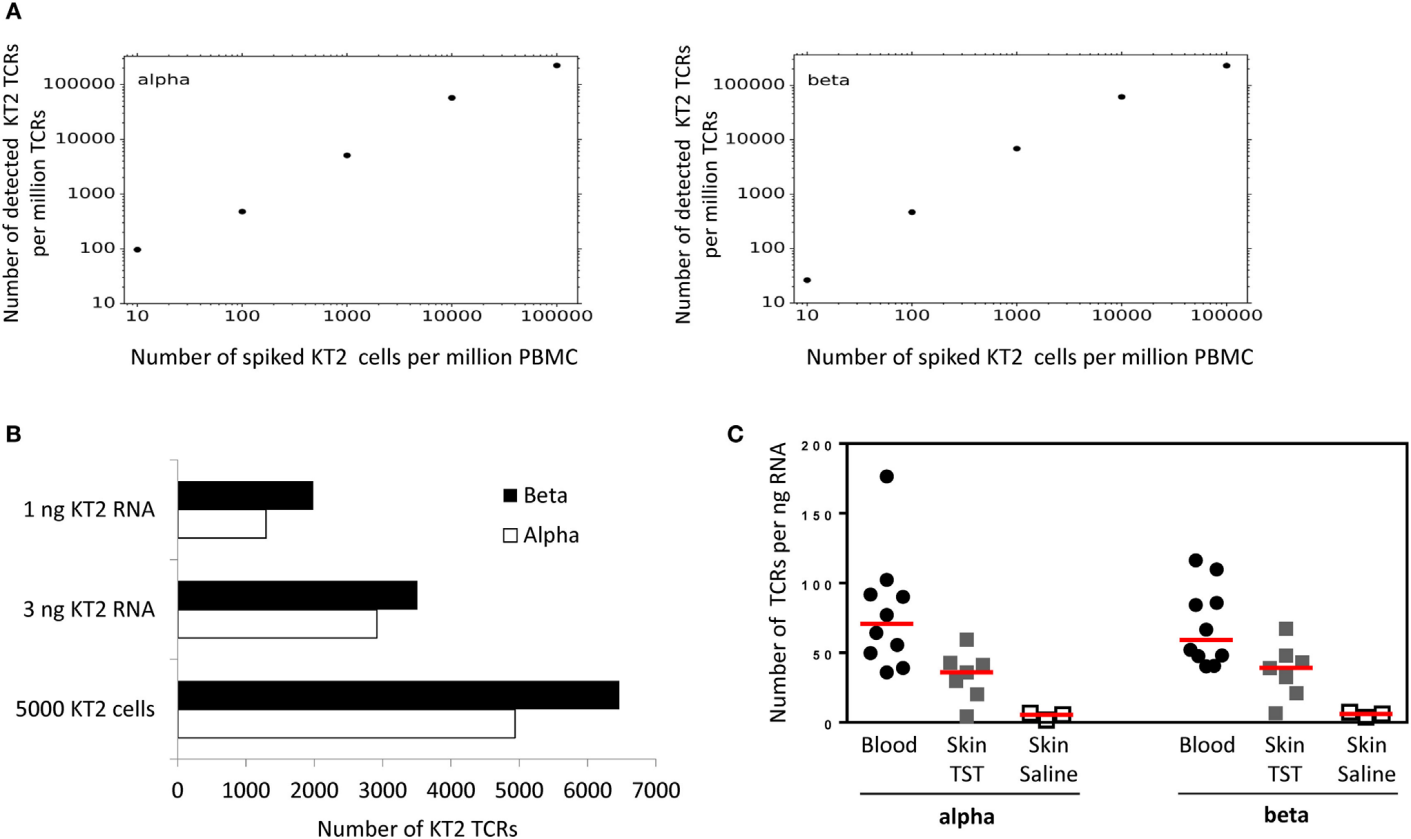
Quantitative analysis of KT2 T cell receptor (TCRs). **(A)** KT2 cells were mixed with a fixed number of peripheral blood mononuclear cell (PBMC) to give a range of KT2:PBMC ratios as shown. The cell mixture was lysed, RNA extracted and TCRs amplified, sequenced, and analyzed as described in the Section “Method.” The figure shows the number of distinct KT2 alpha or beta TCRs identified per million TCRs, as a function of the number of KT cells per million PBMC. A distinct KT2 TCR is defined by a cluster of KT2 TCRs sharing a single unique molecular identifier. **(B)** Top two bars: 5 × 10^5^ KT2 cells were lysed, the total RNA extracted and then the indicated amount of RNA was processed for TCR sequencing. Bottom bars: 5 × 10^3^ KT2 cells were lysed, the total RNA extracted and processed for TCR sequencing. The bar chart indicates the number of distinct KT2 sequences identified from each sample. **(C)** Number of TCR alpha and beta sequences (after error correction) from whole blood or 3-mm skin punch biopsies. Data are expressed as total number of TCR sequences normalized against input RNA amount data points represent individual participants, and group medians are indicated by red lines [Blood, *n* = 10; tuberculin skin test (TST), *n* = 7; Saline, *n* = 3].

The sensitivity and accuracy of the pipeline were tested by “spiking” a population of unfractionated PBMC with known numbers of a T cell clone, KT2, whose TCR had previously been sequenced. The results are shown in Figure 4. The numbers of KT2 alpha and beta sequences were a linear function of the input number of cells over several orders of magnitude, showing evidence of saturation only at very high numbers of KT2. The analysis was able to detect KT2 sequences down to the lowest number tested (1 in 10^5^).

The total amount of input RNA in the spike-in experiment shown in Figure 4 was kept constant. In order to explore the sensitivity of the method using different amounts of input RNA or cells, we made libraries using either low numbers of cells (5,000 KT2 cells), or from very low amounts of RNA (Figure 4B). The number of TCR sequences detected (after UMI-based error correction) was in the same order of magnitude as the number of cells, suggesting the method was able to detect on average at least one transcript from every cell, even at low cell numbers. In order to explore the relationship between T cell number and TCR mRNA molecules further, we sequenced six samples of PBMC from healthy volunteers, in which the number of T cells was determined by flow cytometry. The average (±SEM) ratio of UMI: T cell for these samples was 3.8 ± 1 for TCRα and 3.3 ± 0.6 for TCRβ.

Finally, in order to explore the robustness of the pipeline in samples where T cells are a minority population, we made libraries from RNA extracted from skin biopsies from sites injected with saline (sampling the resident skin T cell population) or injected with PPD in individuals showing a positive Mantoux test. TCR sequences could be recovered from both samples, with the large increase in number of TCRs in the latter reflecting the T cell infiltration associated with a positive delayed type IV hypersensitivity reaction (Figure 4C).

### The TCR Repertoire of Human PB Naïve and Memory Subpopulations

We used the pipeline to compare the population structure of human naïve and memory T cell populations. Although some previous studies have analyzed this question, differing reports have been published (31–34). In part, this reflects the fact that most previous studies have fractionated PB T cells based only on the CD45 isotypes, even though CD45RA+ memory cell populations are well described. In addition, many previous studies have not used UMI-based error correction. We fractionated cells from three healthy volunteers into CD4 and CD8 T cells, and then fractionated each of these populations into four subpopulations based on a combination of CD45RA isoform, and CD27 expression (Figure S5 in Supplementary Material). This combination of markers has been used extensively to characterize naïve (CD45RA+ high, CD27+), central memory (CM, CD45RA−, CD27+), effector memory (EM, CD45RA−, CD27−), and effector memory RA+ (EMRA:CD45RA+, CD27−) (35) T cells.

**Figure 5:**
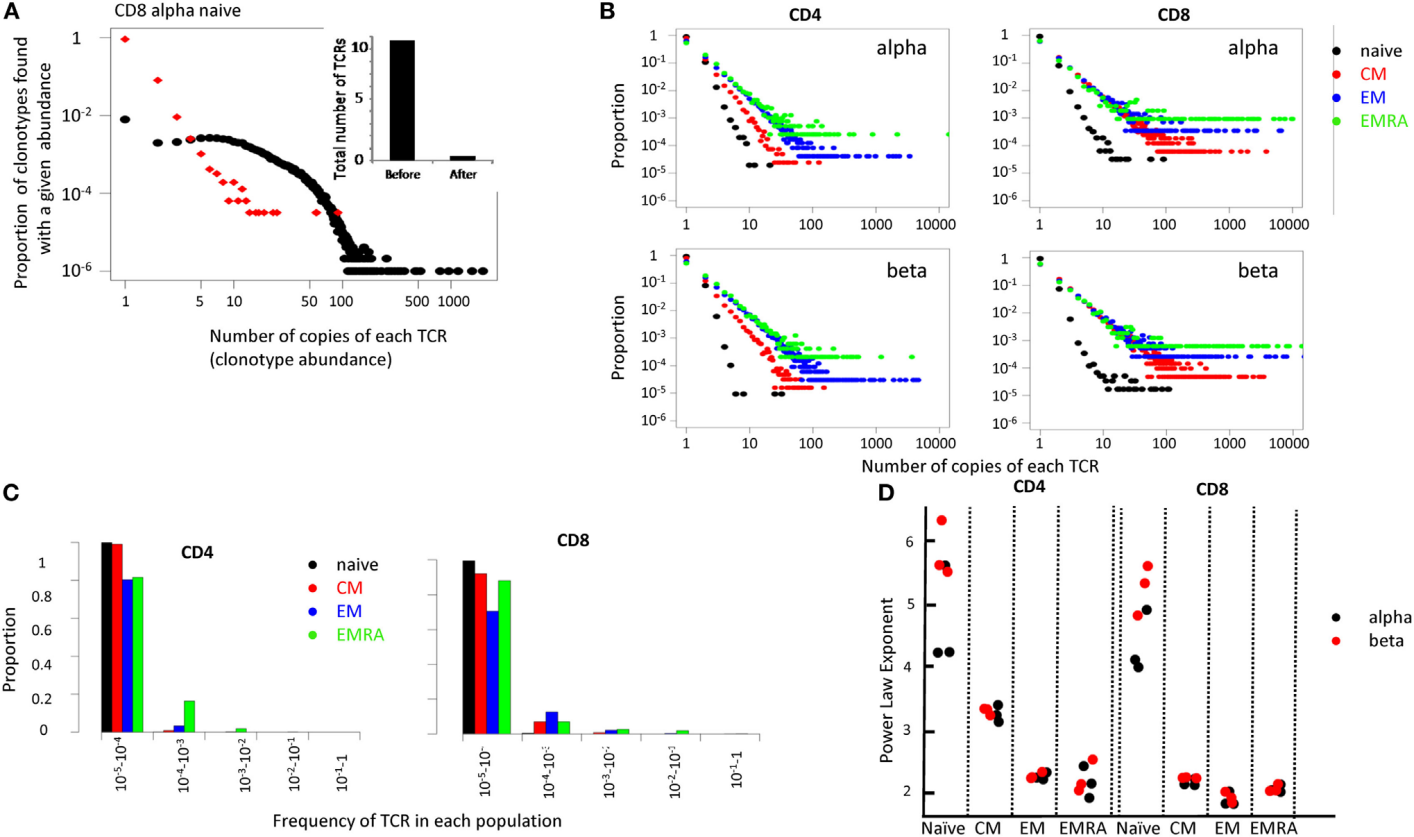
**(A)** The distribution of T cell receptor (TCR) abundances before and after error correction from the naive CD8 repertoire of one representative individual. Inset shows total number of distinct cDNA TCR sequences ×10^5^, calculated before and after error correction. **(B)** Distribution of TCR clonotype size (the proportion of TCR alpha sequences which occur once, twice, etc.) for all subsets. **(C)** A bar plot showing the proportion of TCRs within different frequency ranges for each population. **(D)** The power law exponent for all subpopulations of CD4 and CD8 repertoires from three healthy volunteers (all HLA-A02 positive).

In order to capture the overall population structure of the repertoires of these distinct populations, we plotted the proportion of TCRs that were present once, twice, etc. (Figure 5). Although strictly speaking, the term clonotype refers to an alpha/beta pair, for convenience we use the term to refer to each distinct TCR alpha or beta sequence. We refer to the number of times each such clonotype is found in a repertoire as the clonotype abundance. We first explored the effect of the UMI-based error correction on the clonotype abundance distribution (Figure 5A shows the results for TCR alphas from one naïve CD8 sample, Figure S6 in Supplementary Material compares the effect on a naive and a memory CD4 and CD8 repertoire). As expected, the total number of distinct clonotypes, the maximum TCR abundance observed, and the number of TCRs with large abundances (>100) was decreased following error correction. We compared the results of our analysis pipeline on this sample to an analysis of the same sample using another UMI-based analysis tool, MIGEC (11). Both the total number of UMIs detected, the number of unique TCRs and the clonal distribution of the corrected data, were similar for the two methods (see Table S4 and Figure S7 in Supplementary Material).

The error-corrected distributions of the proportion of TCRs with given alpha and beta clonotype abundance for naïve, CM, EM, and EMRA CD4+ and CD8+ populations from one representative individual are shown in Figure 5B, and the data are summarized in histogram form in Figure 5C. The distributions for alpha and beta chains in each case were very similar, giving some confidence that the TCR sequence numbers reflect the true underlying cellular distribution and not some feature of the library preparation. In each case, the distribution of the naïve population had a much steeper slope, with >95% TCR sequences appearing only once. This is reflected by the fact that almost all naïve TCRs are present at a frequency of less than 1 in 10^4^ (Figure 5C). By contrast, over 20% of all the memory TCRs are present at a frequency of greater than 1 in 10^4^ (Figure 5C). The distributions on the log–log plots (Figure 5B) appear to be linear for all except a few of the largest clones in each distribution. This linear pattern is characteristic of power law distributions [*f*(*x*) = *ax*^−^*^b^*], which have frequently been linked to TCR repertoire distributions previously (36–39). We, therefore, fitted the data within the linear range to a power law equation, using maximum likelihood estimators as described (40, 41). The fitted plots for one individuals’ CD4 and CD8 populations are shown in Figure S8 in Supplementary Material. The exponent of the fitted power law for all subpopulations for three different individuals are shown in Figure 5D.

The estimated slopes (the power law exponent) were consistent between all three individuals. The naïve population had a very steep slope (exponent >3), with almost all the TCRs present less than 10 times. The estimated parameter values for the naïve also showed the biggest variance, reflecting the uncertainty in fitting a small number of data points, lying on a very steep slope. For CD4 cells, CM consistently showed a larger exponent than EM and EMRAs (*T*-test *p* < 0.01). By contrast, for CD8 cells the slope for CM, EM, and EMRA were very similar (*p* > 0.05).

**Figure 6:**
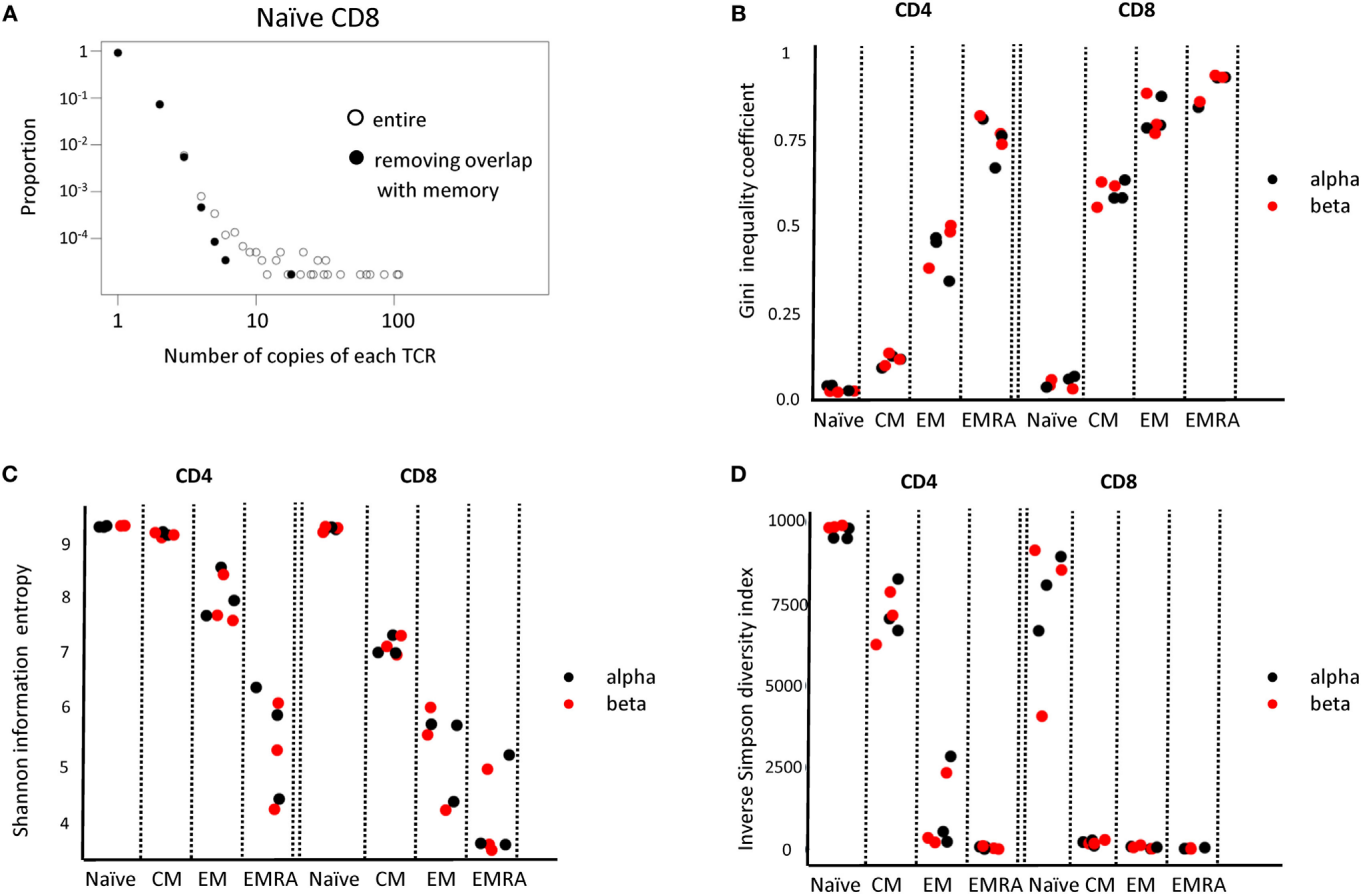
**(A)** The distribution of T cell receptor (TCR) beta clonotype abundances in the naive CD8 population (one representative individual from three) before and after removing all those TCRs which are also found within one of the memory populations. **(B–D)** The Gini coefficient, the Shannon entropy and the Inverse Simpson diversity index calculated for each CD4 and CD8 subpopulation, for each of three individuals. All repertoires are subsampled to the same number of TCRs before calculating the index.

Above a certain limit, all distributions were over-dispersed, with a small number of TCRs present at very large abundances. In part, this reflects the fact that the plots contain a lower frequency limit which is determined by the size of the sample; clonotypes cannot occur less than once in the sample. The plot is, therefore, truncated at the lowest observable frequency which is given by 1/sample size. However, we noted that even naïve repertoires, especially in the CD8 population, contained a few larger clonotypes. These clones might reflect memory cells which re-express CD45RA (42, 43). We, therefore, removed from the set of naïve TCRs any sequence which was also found in one or more of the memory subpopulations from the same donor. This removed almost all large clones from the naïve repertoires (Figure 6A).

We next calculated a number of TCR repertoire population parameters that have been used previously in the characterization of TCR repertoires (44). Since these diversity indices are affected by population size, we subsampled each repertoire to a fixed number of UMI-defined distinct TCR mRNA molecules. The Gini index, which captures the inequality in clonotype size across the population is shown in Figure 6B. As predicted, both CD4 and CD8 naïve T cells have a very low Gini index (naïve < all other groups for both CD4 and CD8, *p* < 0.01, Student’s *t*-test, *n* = 6). The index increases as one examines the CM, EM, and EMRA subpopulations, reflecting the emergence of more highly expanded clones. The Gini index for the alpha and beta chain is similar. The Shannon Information Entropy and the Inverse Simpson index (Figures 6C,D), frequently used as measures of repertoire diversity, shows the inverse pattern, decreasing with the progression from naïve through CM, EM, and EMRA (naïve > all other groups for both CD4 and CD8, *p* < 0.01, Student’s *t*-test, *n* = 6).

**Figure 7:**
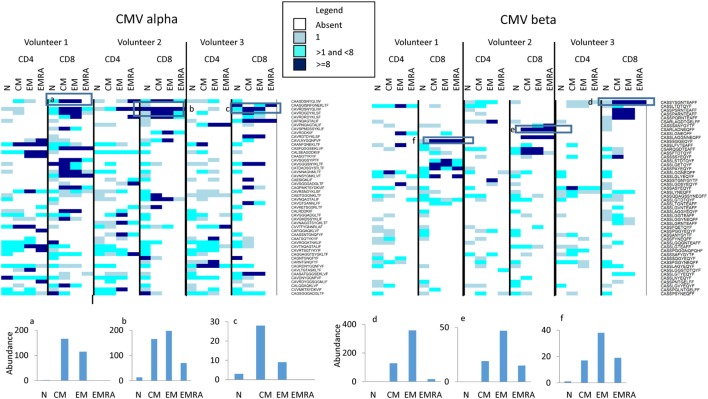
The abundance of CMV-associated CDR3s in naïve and memory populations. The heatmap shows the number of times (see Legend for color code) each CDR3 sequence (shown on right of heatmap) is found in the respective population (along top of heatmap). For clarity, only the 50 CDR3 with the highest abundance are shown. The actual abundance for six highly expressed CDR3s (those enclosed in a square box in the heatmap) is shown in the histograms shown below the heatmaps.

Finally, we examined whether we could detect evidence of antigen specific responses in these data sets. We downloaded the full set of annotated CMV-specific CDR3 sequences from the public repository VDJdb^4^ and from Chen et al (45), a total of 7,322 distinct CDR3 sequences. We searched for these in the naïve and memory repertories from the three individuals described above (Figure 7). 2,375 TCRα and 1,010 TCRβ sequences were found at least once in our three individuals. The majority of were present only as sporadic single sequences in any repertoire. However, a number of expanded CMV-associated CDR3s were observed. These CDR3s were generally absent or present only at low frequencies in naïve populations, but were found expanded in one or more memory populations (see histograms at bottom of Figure 7). Both private and public CDR3s were observed, although expansion of the public CDR3s was not necessarily the same in all individuals in which the CDR3 was present. Volunteer 2 was CMV negative as measured by functional T cell responses, but expanded CDR3s were observed in this individual too.

## Discussion

This paper describes an integrated experimental and analytical pipeline for the study of the T cell repertoire. As a practical example, we apply the pipeline to compare the properties of naïve and memory subcompartments in human PB T cells. Since the advent of next generation massively parallel sequencing, a number of experimental (6) and computational (8) protocols for the analysis of TCR repertoire have been reported. A side-by side comparison of all these different techniques would be challenging at both cost and complexity levels for any one laboratory. A collaborative project between a set of different laboratories to carry out such comparisons on a single shared RNA sample is currently in progress, but lies outside the scope of this publication. Furthermore, it is difficult to compare like-for-like, since some protocols use DNA, some RNA, and some have proprietary elements which cannot be readily reproduced. Instead, we highlight the major differences between our methods and some of the others which have been reported in the literature.

The first major feature of our protocol is that both experimental and computational methods reported here are open source, and details of all reagents and information are freely available to the community. The majority of published high-throughput investigations of T cell repertoires outsource the TCR sequencing to commercial service providers, and the full details of the methods and the raw sequence data are frequently not available. This makes analysis of the reproducibility of such studies, or comparison with other results, very difficult. This difficulty is compounded by the fact that commercial protocols do not generally incorporate UMI’s and rely on undisclosed algorithms which correct for potential PCR bias (see below).

Perhaps the most important technical feature of the protocol described above is the incorporation of UMIs, in which an oligonucleotide containing a random dodecamer sequence is ligated to the 3′ end of each cDNA molecule. The importance of such an identifier has been emphasized previously (46, 47). In particular, it facilitates two types of corrections. The first is corrections for PCR and sequencing errors, which can be largely identified by comparison of TCRs with the same or very similar UMI. The second is correction for bias introduced by differences in PCR efficiency which, as we have shown previously (13), is an intrinsic feature of all PCR and would otherwise greatly distort the frequency distributions of the population. Quantitative conclusions about TCR frequencies can be influenced by these corrections, and in some cases may influence the conclusions of such studies which do not include UMIs. This is likely to be particularly true for the less frequent TCRs, which will comprise the majority of unique sequences for almost all physiologically relevant T cell populations (see Figure S6 in Supplementary Material). For example, the power law distribution reported here and previously would be totally obscured without UMI correction. The quantitative behavior of the pipeline reported here is supported by the results of the spike-in experiments using the monoclonal line KT2, which accurately detected specific TCRs within a large mixed pool over a broad range of clonotype abundance.

Another important aspect of our study was to develop an assay to measure the efficiency of the key ligation step. T4 RNA ligase has been widely used for single-stranded DNA ligation in the context of RACE protocols since its introduction for library preparation nearly three decades ago (19, 20). Although the ligase is often anecdotally described as having a low efficiency when ligating DNA substrate, no measurements of efficiency have been published. In fact, we found the efficiency of the enzyme to be at least 10%, which is significantly higher than the reported efficiency of template switching RT-PCR, a commonly used alternative technique for introducing UMIs into TCR sequencing (11, 12). The efficiency of the first few steps of library preparation, prior to any PCR amplification step, is crucial in the coverage of the library. Any molecules of cDNA which are not recovered during this step will be permanently lost from the final library. By contrast, once the cDNA molecules have gone through a few amplification cycles, the likelihood of losing all copies of a TCR are much reduced. The presence of multiple copies of each TCR mRNA within a T cell is advantageous in this regard, since it decreases the probability of all molecules from one cell being lost following lysis. As an example, one may consider a typical RNA sample prepared from one million cells. Based on qPCR results, we estimate that both the first two purification steps in the protocol (PU1 and PU2 in Figure 1) may each result in recoveries of only 50% of the DNA from the preceding step (partly because the concentration of specific cDNA is very low at this point). Combined with a 10% recovery at the ligation step, this results in an overall recovery of 2.5% of the original input cDNA prior to PCR1. On the basis of 100 TCR mRNA molecules per cell, this would result in 1–3 molecules from each cell entering the amplification step of the procedure, resulting in almost complete coverage of the sample repertoire. Further work is in progress to more accurately estimate likely repertoire coverage by computational simulations of the whole pipeline.

As a practical example, the technology is applied to an analysis of the TCR repertoires of different subpopulations of human PB T cells. After the UMI-based correction process, the different subpopulations isolated from three different individuals show some very characteristic properties. The most striking differences are seen between naïve (defined as CD45RA+ high CD27+) and memory populations. The clone size distribution of naïve CD4 and CD8 populations are almost identical, with very steep gradients and with over 95% of TCRs observed only once in each sample, and only a very few sequences observed more than five times. The overwhelming presence of singletons or very small sets of identical TCRs is reflected in a very low Gini index, and a very high Shannon entropy and Simpsons’ diversity index. By contrast, all the memory populations contained many more larger sets of identical TCRs and, hence, showed a larger Gini index and lower diversity indices. When comparing the different subpopulations of memory cells, the picture becomes more complex. In general, the parameters of the TCR repertoire distribution of CM cells showed the most similarity to naïve cells, followed by EM and then EMRA, consistent with the “self-renewing effector model” of memory T cell differentiation (48) as discussed further below.

Previous studies of human T cell naïve and memory subpopulations have given inconsistent results (31, 32, 49, 50), and in evaluating the results of our current analyses there is no “ground truth” to which we can compare our findings. Nevertheless, the results are at least compatible with current thinking about the T cell compartment. For example, the naïve cell repertoire is considered to be generated by a large number of diverse clones of small size (less than 100 cells) generated during intra-thymic selection and expansion. Against this background, naïve clone size heterogeneity can also develop, driven by clonal competition for self-antigens (51, 52). This may perhaps explain the small number of more abundant TCRs we observed within the naïve population, even after removing from the naïve TCR populations those sequences that were also observed in memory compartments from the analysis. The gradual increase in TCR abundance, increase in Gini and fall in Shannon entropy as one progresses from naive through CM, EM, and EMRA compartments is also compatible with the process of antigen-driven clonal expansion, and specifically with the model of sequential differentiation of cells into CM, EM, and EMRA proposed on the basis of single-cell tracking experiments in mice (53). In this context, it was notable that HLA-A*02 CMV-associated CDR3s were observed in both HLA-2 CMV positive individuals analyzed, and in each case the same CDR3 was expanded in CM, EM, and in the majority of instances in EMRA populations as well. The T cells expanded by chronic exposure to this virus, therefore, populate all subpopulations of the memory compartment (54).

In summary, we report the details of a new protocol for the amplification and sequencing of a UMI-labeled cDNA TCR library, together with a set of software modules for the subsequent analysis. We believe that the simplicity and flexibility of the pipeline, its relative low cost, and its incorporation of a UMI-based correction pipeline will make the pipeline attractive to the growing number of researchers interested in exploring the TCR repertoire in various clinical preclinical settings. We hope that the publication of all protocol details, the sharing of key reagents and sequences, and creation of a repository containing all open source scripts will facilitate the development of a community of users. Interaction between members of such a community will further improve and validate the methods making them a useful resource for the entire immunological community.

## Ethics Statement

This study was carried out in accordance with the recommendations of the UK Research Ethics Committee with written informed consent from all subjects. All subjects gave written informed consent in accordance with the Declaration of Helsinki. The protocol was approved by the University College London Hospital Ethics Committee 06/Q0502/92.

## Author Contributions

TO and JH contributed equally to this work. BC initiated and managed the study and drafted MS. TO, JH, CH, and KJ contributed to developing the wetlab protocols. TR and IU contributed to measuring TCR transcript abundance. BC carried out the experiments shown in Figure 2. JH, KB, and MI contributed to developing the computation pipeline. TO and NR performed the experiments shown in Figures 5–7. TO, CT, RB-M, and MN carried out the experiments in Figure 4. GM contributed to managing the overall project. All authors commented and revised the MS.

## Conflict of Interest Statement

The authors declare that the research was conducted in the absence of any commercial or financial relationships that could be construed as a potential conflict of interest.

## Footnotes

^1^https://github.com/innate2adaptive/automate-decombinator.

^2^https://support.illumina.com/downloads/bcl2fastq_conversion_software_184.html.

^3^https://www.ncbi.nlm.nih.gov/sra.

^4^https://vdjdb.cdr3.net/.
