# Supplementary material for: Quantitative Characterization of the T Cell Receptor Repertoire of Naïve and Memory Subsets Using an Integrated Experimental and Computational Pipeline Which Is Robust, Economical, and Versatile
Source: Front Immunol. 2017 Oct 12;8:1267. doi: 10.3389/fimmu.2017.01267 (PMC5643411; doi:10.3389/fimmu.2017.01267)

Supplementary figures legends

- Supplementary Figure 1. PCR2 (a) and tape station electrophoresis (b) for alpha and beta chains processed from 10^6 and 10^5 KT2 cells as shown in Table. a) A quantitative SybrGreen qPCR was performed. The samples crossed the manually set threshold of 0.1ΔRn after 12-14 cycles. The PCR reaction was stopped after 15 cycles. The TCR alpha and TCR beta controls crossed the threshold after ~ 11 cycles.
- Supplementary Figure 2. The influence of read depth on the number of distinct UMI-TCR combinations detected in a sample. The repertoire of three samples of healthy volunteer PB was sequenced as described. The resulting Fastq files (each sample contained 1-2 million reads) were randomly sampled to different depth, and then analysed using Decombinator followed by Collapsinator. The number of distinct UMI-TCR s detected is plotted against the proportion of sequences analysed.
- Supplementary Figure 3. A frequency histogram showing the number of times distinct barcodes are sequenced. The 8 panels correspond to different subpopulations of peripheral blood T cells from one representative individual as shown in supplementary fig 5, and figs 5 and 6.
- Supplementary Figure 4. a) Example of standard curves obtained by amplifying plasmids containing alpha or beta constant region sequences. The plots show the cycle value at which the amplification curves hit the threshold. b) Representative example of real time amplification data for the standard curve shown in a. Each point is done in duplicate.
- Supplementary Figure 5. Gating strategy for subfractionation by FACS sorting of the CD4 naïve and memory populations from one individual (see Fig 5 -7).
- Supplementary Figure 6. CDR3 clone size in effector memory and naïve T cell populations: The size of each CDR3 before and after correction for sequencing error/PCR amplification (Collapsing) using UMIs. CDR3 family sizes are larger before than after Collapsing (found to the left of the line of symmetry).
- Supplementary Figure 7. The clonotype abundance frequency distribution for a representative CD8 naive population analysed using the analysis pipeline described in this paper (Decombinator followed by Collapsinator, open circles) and analysed using MIGEC (filled diamonds).
- Supplementary Figure 8. The clonotype abundance frequency distribution for all subpopulations from one of the three individuals analysed. The dots show the actual data, and the solid lines show the power law distribution of best fit, over the range shown, using maximum likelihood estimation.

Supplementary figure 1

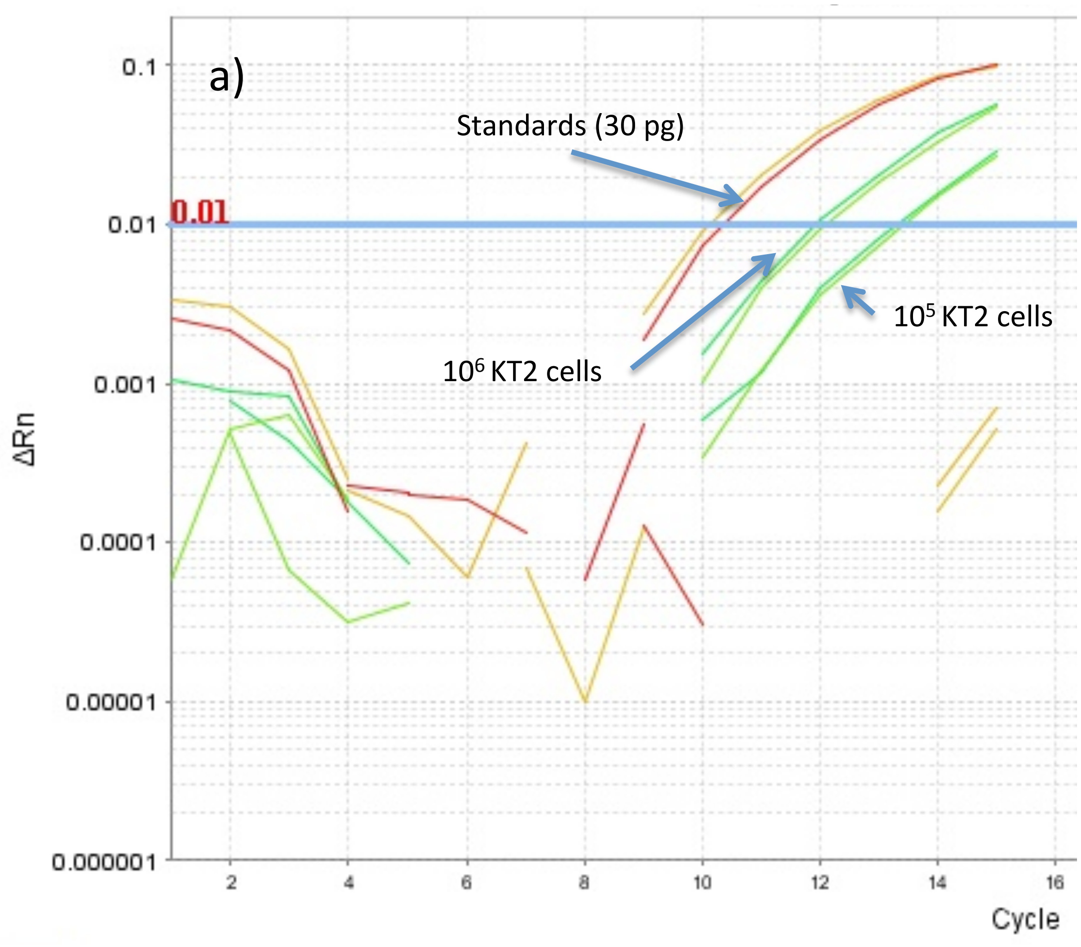

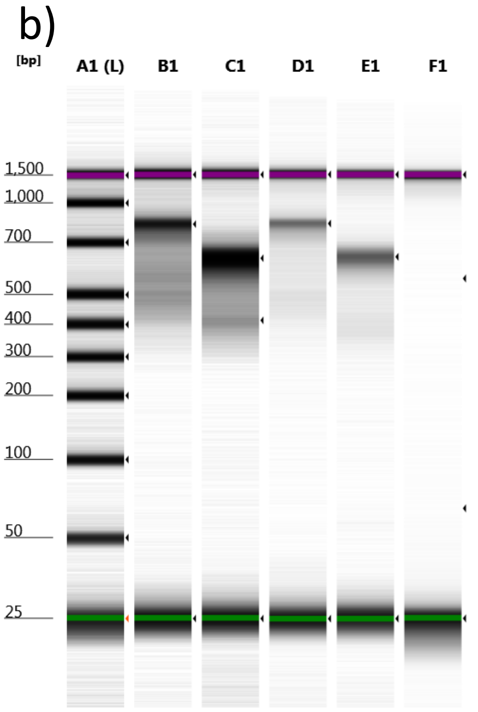

Well	Conc. [pg/µl]	Sample Description
A1	1800	Ladder
B1	307	1x10^6 alpha
C1	768	1x10^6 beta
D1	140	1x10^5 alpha
E1	220	1x10^5 beta
F1	2.95	empty

Supplementary figure 2

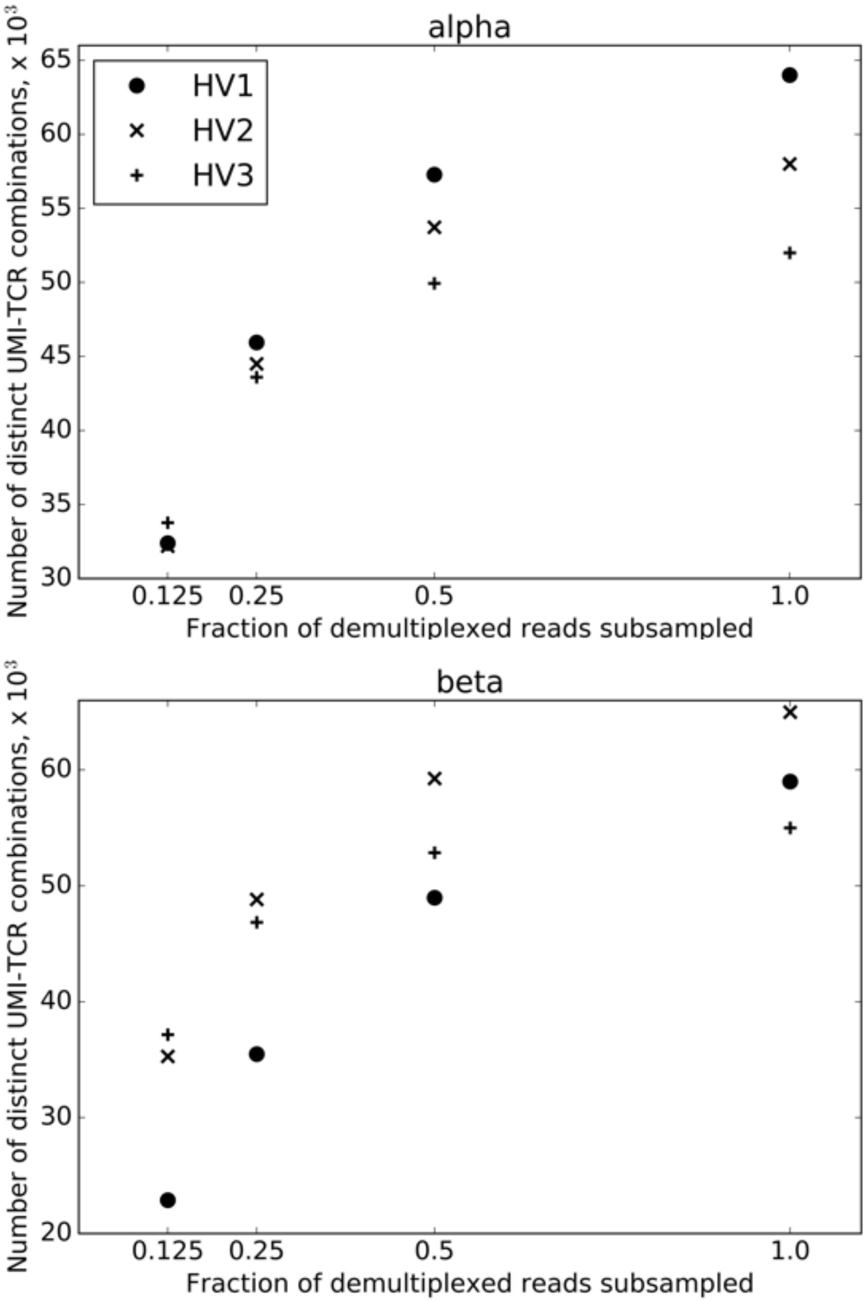

Supplementary figure 3

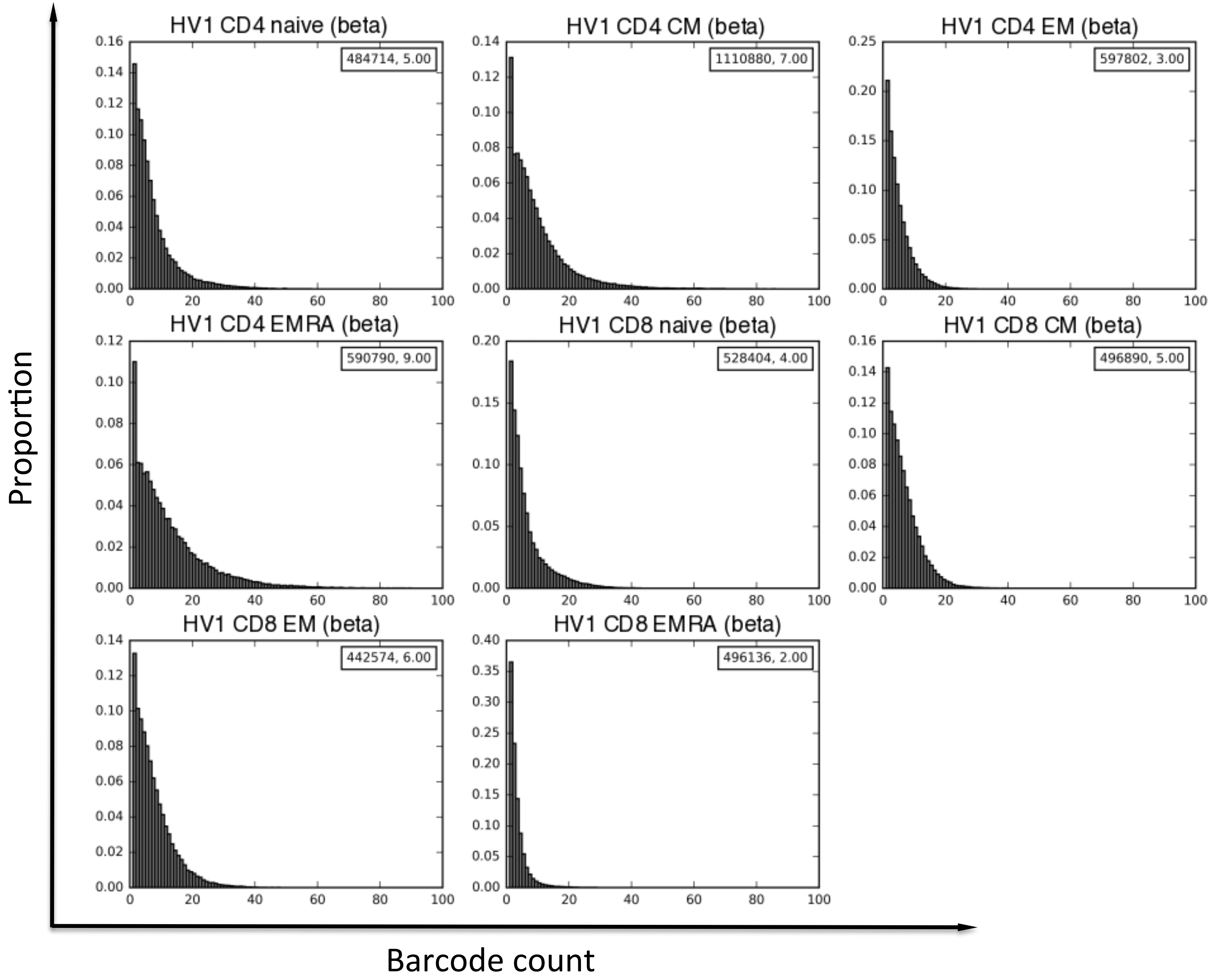

Supplementary figure 4

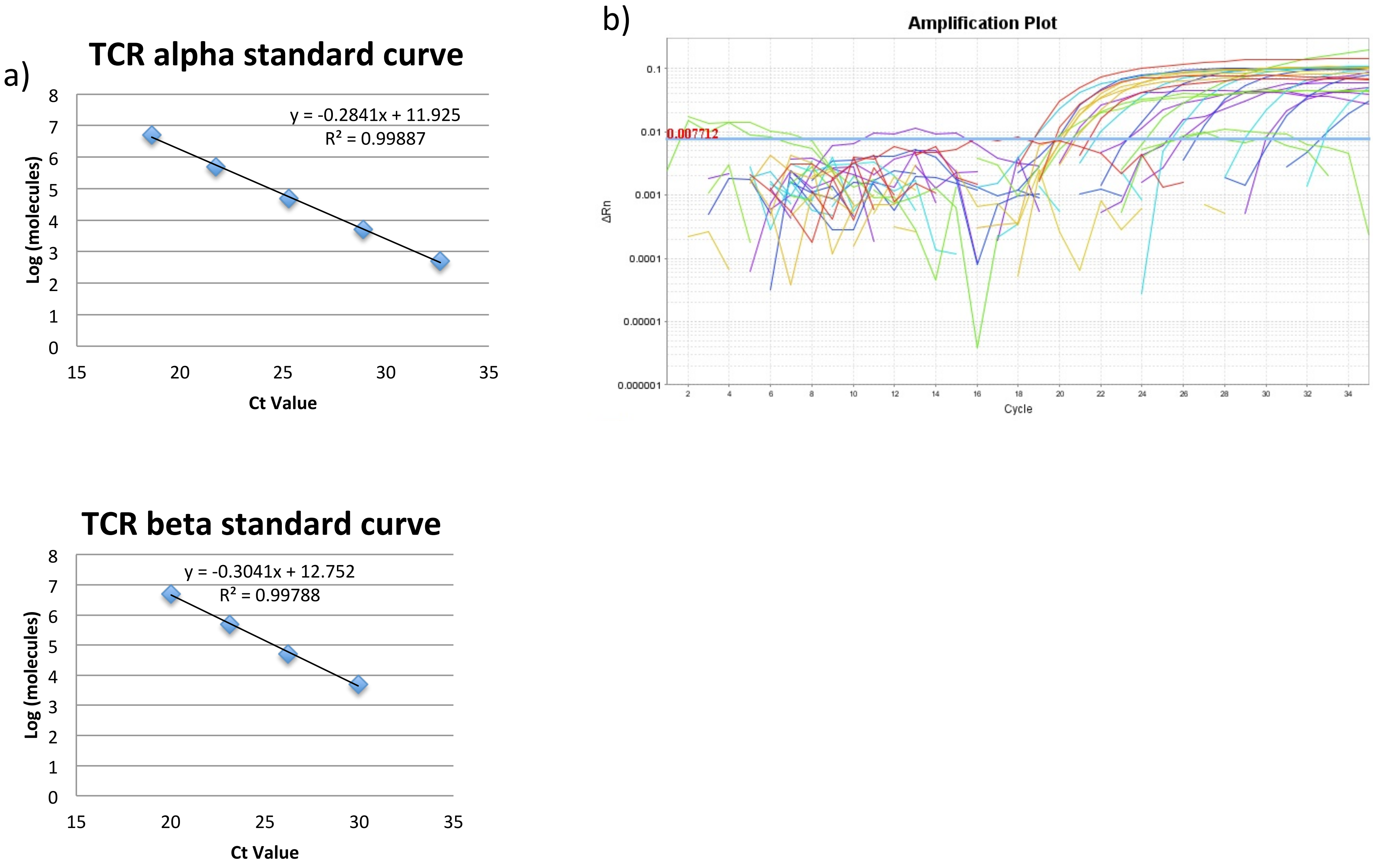

Supplementary figure 5

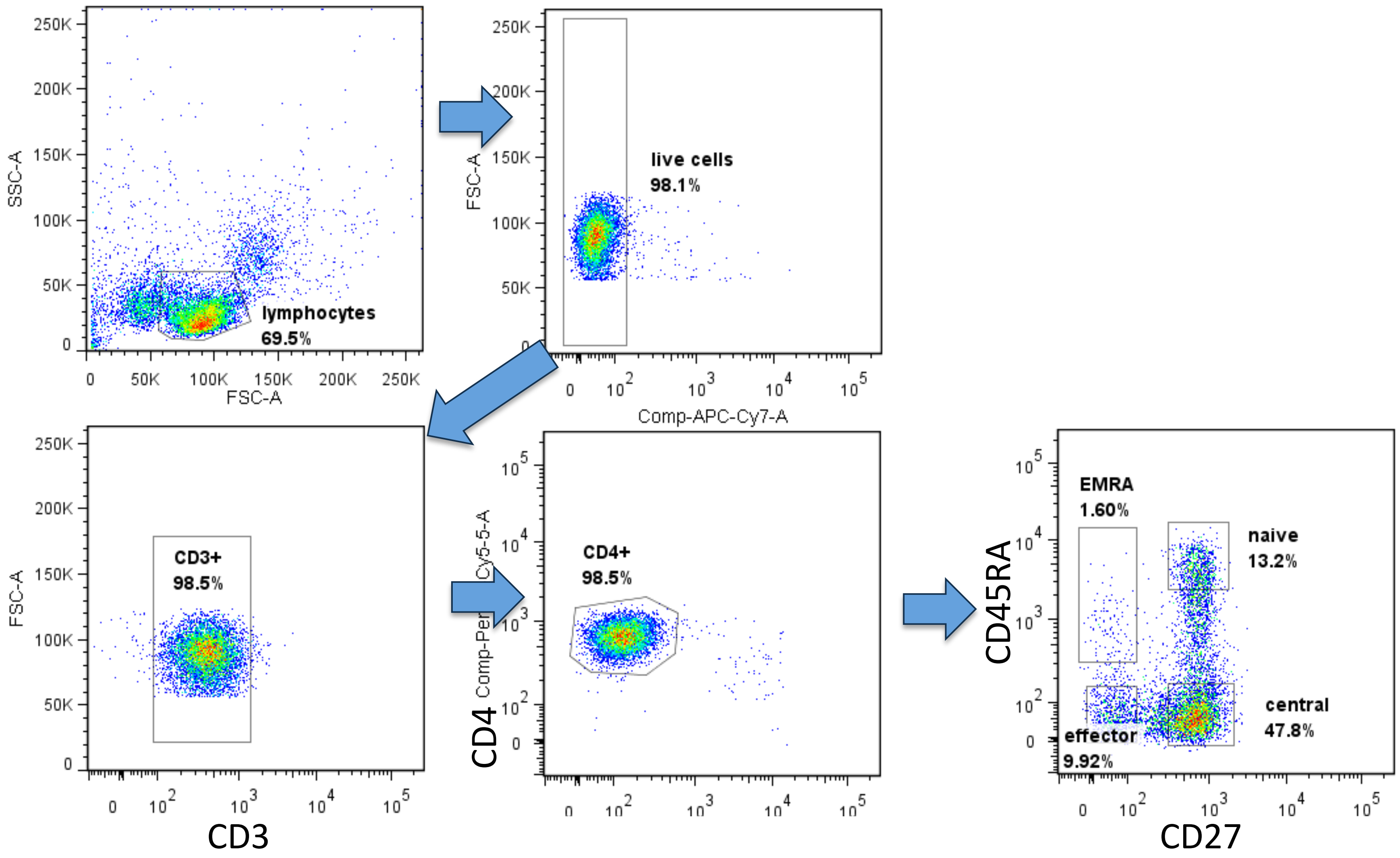

Supplementary figure 6

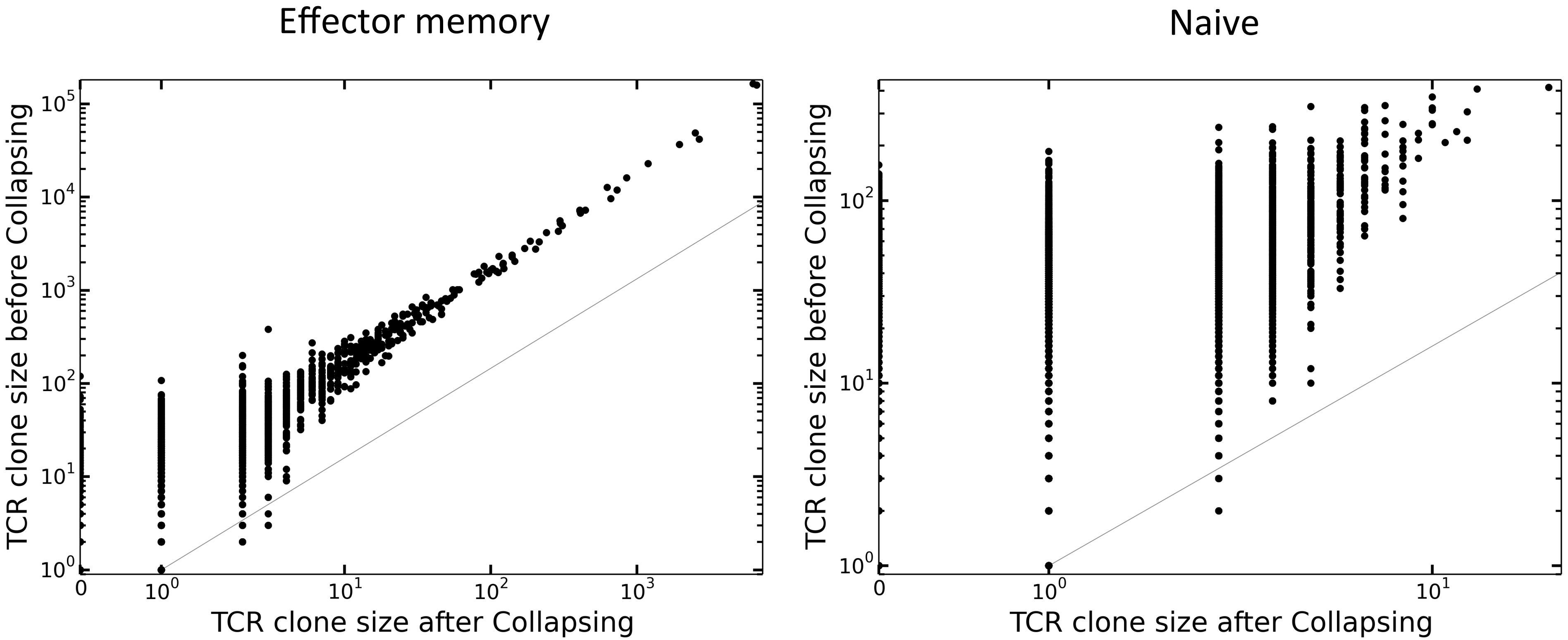

Supplementary figure 7

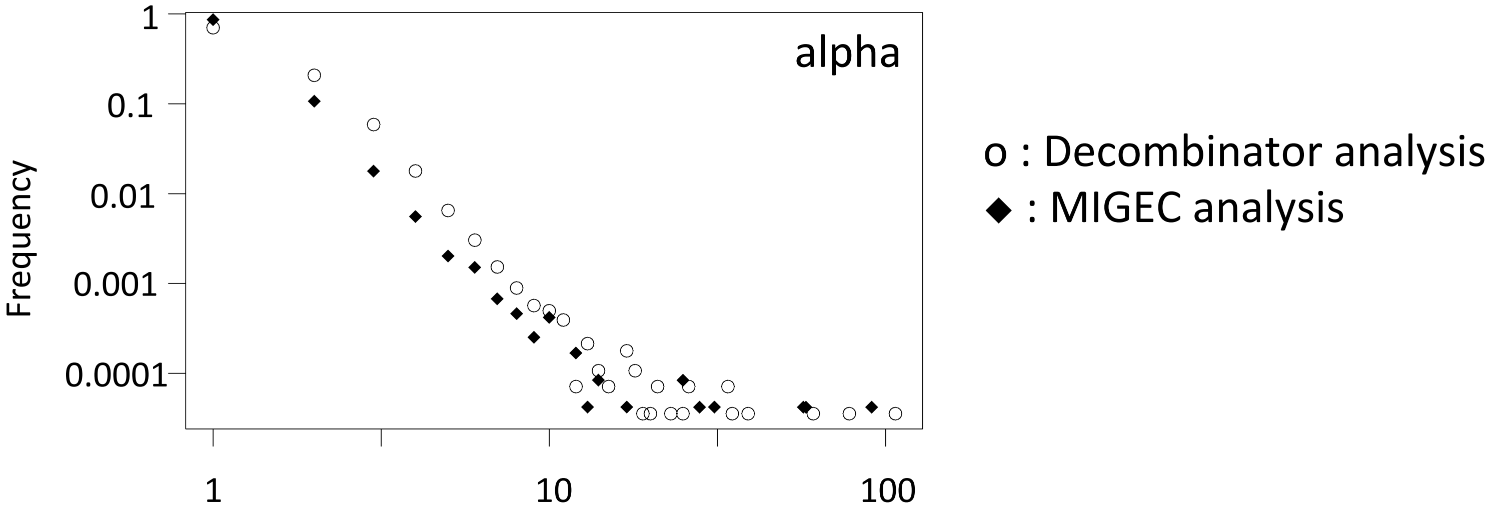

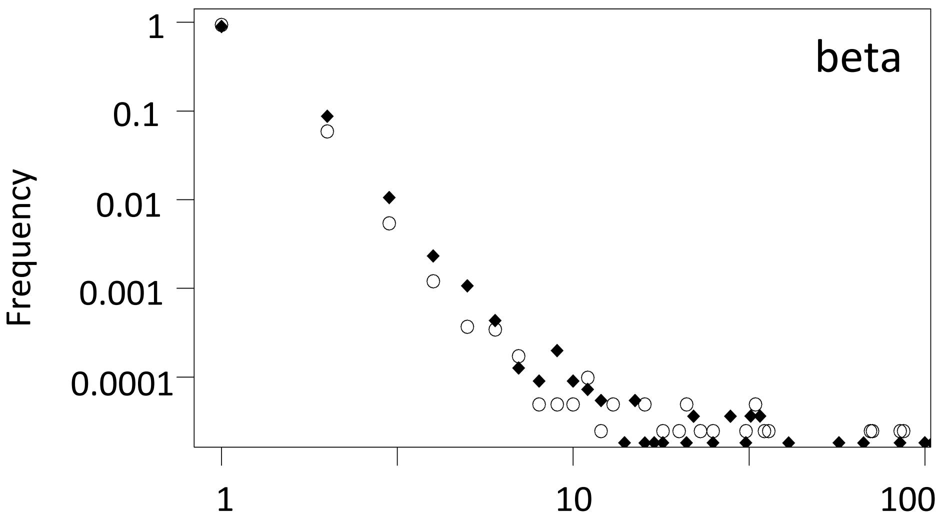

Supplementary figure 8

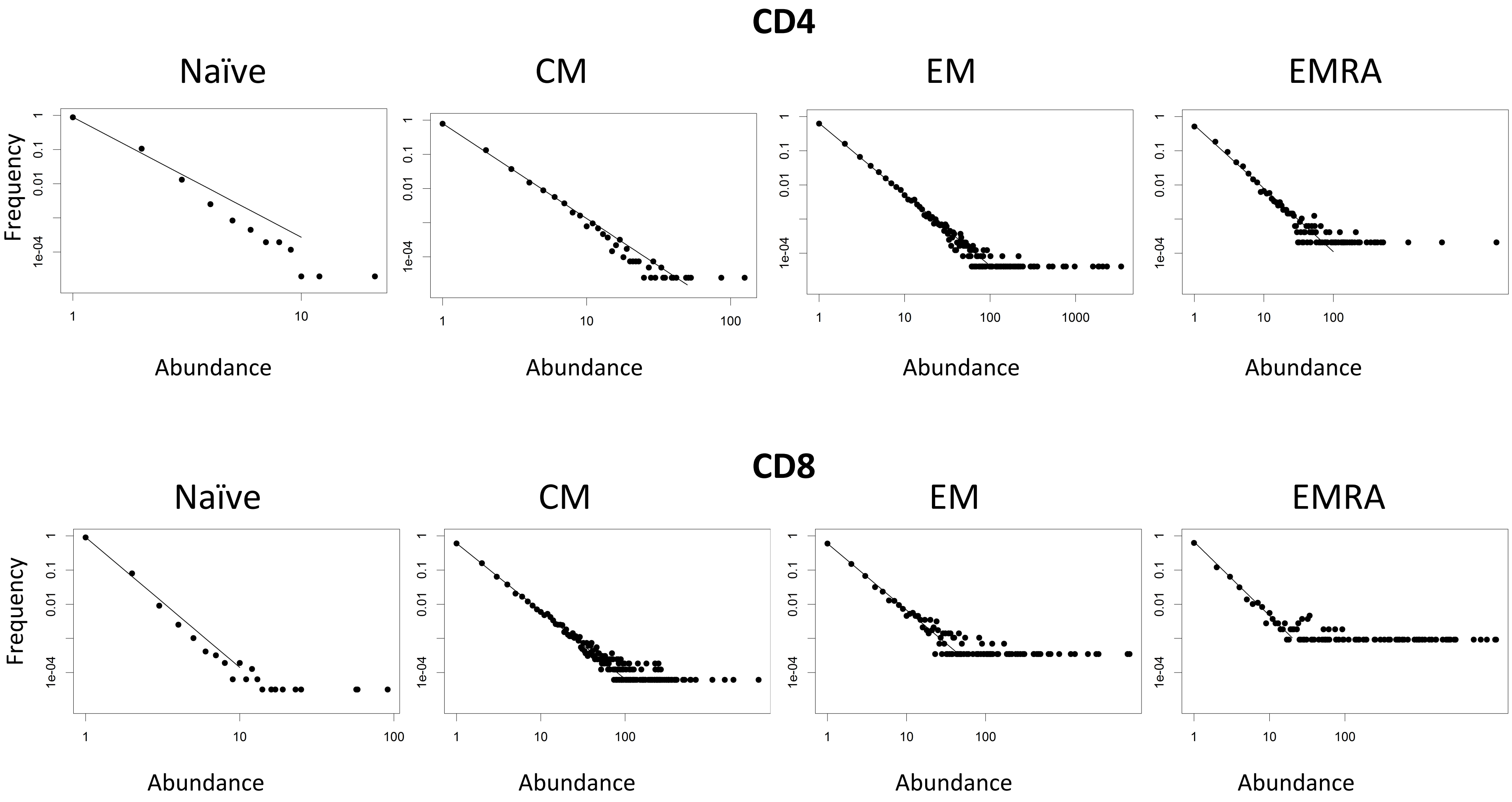

Supplement: Supplementary file 1 [file presentation_1.pdf]
