# Supplementary material for: Quantitative Characterization of the T Cell Receptor Repertoire of Naïve and Memory Subsets Using an Integrated Experimental and Computational Pipeline Which Is Robust, Economical, and Versatile
Source: Front Immunol. 2017 Oct 12;8:1267. doi: 10.3389/fimmu.2017.01267 (PMC5643411; doi:10.3389/fimmu.2017.01267)
Supplement: Supplementary file 2 [file data_sheet_1.pdf]

Supplementary Table 1 Sample details for the cell sorting and RNA extraction of naïve and memory subpopulations as described in Methods, and shown in Supplementary Figure 5.

Sample ID	Sample Name	Donor	cell number	RNA				HLA-A and CMV status
				Conc (ng/(µl)	260/280	260/230	Volume (µl)	
4000	CD4+CD27+ RA+ naïve	SK11	1.82x10^6	109.2	NT	NT	40	A2+ CMV+
4001	CD4+CD27+ RA- CM	SK11	8.7x10^6	209	NT	NT	40	A2+ CMV+
4002	CD4+CD27- RA- EM	SK11	1.54x10^6	103.1	NT	NT	40	A2+ CMV+
4003	CD4+ CD27- RA+ EMRA	SK11	2.31x10^5	13.7	NT	NT	40	A2+ CMV+
4004	CD8+CD27+ RA+ naïve	SK11	8.4x10^5	66.9	NT	NT	40	A2+ CMV+
4005	CD8+CD27+ RA- CM	SK11	5.6x10^5	37.5	NT	NT	40	A2+ CMV+
4006	CD8+CD27- RA- EM	SK11	4.69x10^5	30.8	NT	NT	40	A2+ CMV+
4007	CD8+ CD27- RA+ EMRA	SK11	2.05x10^6	153.8	NT	NT	40	A2+ CMV+
4008	CD4+CD27+ RA+ naïve	EG10	5.9x10^6	200.5	1.92	1.94	50	A2+ CMV+
4009	CD4+CD27+ RA- CM	EG10	5.2x10^6	134	1.95	2.14	50	A2+ CMV+
4010	CD4+CD27- RA- EM	EG10	2.65x10^6	92.8	1.91	0.4	40	A2+ CMV+
4011	CD4+ CD27- RA+ EMRA	EG10	2.6x10^5	18.9	1.71	0.36	16	A2+ CMV+
4012	CD8+CD27+ RA+ naïve	EG10	4.1x10^6	147.5	1.94	2.03	50	A2+ CMV+
4013	CD8+CD27+ RA- CM	EG10	2.3x10^6	101.9	1.91	2.2	50	A2+ CMV+
4014	CD8+CD27- RA- EM	EG10	1.5x10^6	53.1	1.87	1.76	50	A2+ CMV+
4015	CD8+ CD27- RA+ EMRA	EG10	1.6x10^6	77.5	1.97	0.17	16	A2+ CMV+
4029	CD4+CD27+ RA+ naïve_1	KS07	3.2x10^6	165.3	1.93	2.25	35	A2+ CMV-
4030	CD4+CD27+ RA+ naïve_2	KS07	3.2x10^6	171.6	1.9	1.62	35	A2+ CMV-
4031	CD4+CD27+ RA+ naïve_3	KS07	3.2x10^6	142.8	1.93	2.12	35	A2+ CMV-
4032	CD4+CD27+ RA+ naïve_4	KS07	3.2x10^6	174.5	1.93	1.89	35	A2+ CMV-
4033	CD4+CD27+ RA- CM	KS07	9.7x10^6	123.1	1.96	1.87	65	A2+ CMV-
4034	CD4+CD27- RA- EM	KS07	2.7x10^6	149.2	1.93	2.08	35	A2+ CMV-
4035	CD4+ CD27- RA+ EMRA	KS07	2.13x10^5	30.6	1.88	0.31	17.5	A2+ CMV-
4036	CD8+CD27+ RA+ naïve_1	KS07	1.767x10^6	101.9	1.93	2.12	35	A2+ CMV-
4037	CD8+CD27+ RA+ naïve_2	KS07	1.767x10^6	101.1	1.89	2	35	A2+ CMV-
4038	CD8+CD27+ RA+ naïve_3	KS07	1.767x10^6	87.7	1.92	1.61	35	A2+ CMV-
4039	CD8+CD27+ RA- CM	KS07	2.6x10^6	138.1	1.92	0.84	35	A2+ CMV-
4040	CD8+CD27- RA- EM	KS07	4.6x10^5	37.7	1.89	2.2	17.5	A2+ CMV-
4041	CD8+ CD27- RA+ EMRA	KS07	4.7x10^5	41.4	1.64	0.76	17.5	A2+ CMV-

Supplementary Table 2 Oligonucleotide sequences for TCR pipeline

Name	Sequence	Protocol step	Modification
	**HUMAN**		
Alpha_RC2	GAGTCTCTCAGCTGGTACACG	3	
Beta_RC2	ACACAGCGACCTCGGGTGGGAA	3	
hTRAC_Q_F	ATATCCAGAACCCTGACCC	3	
hTRAC_Q_R	CATGTCTAGCACAGTTTTGTC	3	
hTRAC_Q_Pr	TCTGTCTGCCTATTCACCG	3	5' 6FAM[2]; 3' BHQ1[3]
hTRBC_Q_F	GAGGTCGCTGTGTTTGAGC	3	
hTRBC_Q_R	TCCTTCCCATTCACCCAC	3	
hTRBC_Q_Pr	AGATCTCCCACACCCAAAAG	3	5' 6FAM[2]; 3' BHQ1[3]
6N_I8.1_6N_I8.1_SP2	[4]NNN NNN ATCACGAC NNN NNN ATCACGAC AGA TCG GAA GAG CAC ACG TCT GAA CTC CAG	4	5'phosphate; 3'C3 spacer
Alpha RC1	ATCTACACTACGGCAGGGTCAGGGTTCTGGATAT	7	
Beta RC1.1	TACACTGGTGGGAACACCTTGTTCAGGTCCTC	7	
Beta RC1.2	ATCTACACTGGTGGGAACACGTTTTTCAGGTCCTC	7	
SP1-6N-I-1-aRC1[5]	ACACTCTTTCCCTACACGACGCTCTTCCGATCTNNNNNNATCACGACGGCAGGGTCAGGGTTCTGGATAT		
SP1-6N-I-1-bRC1.1[5]	ACACTCTTTCCCTACACGACGCTCTTCCGATCTNNNNNNATCACGGGTGGGAACACCTTGTTCAGGTCCTC	7	
SP1-6N-I-1-bRC1.2[5]	ACACTCTTTCCCTACACGACGCTCTTCCGATCTNNNNNNATCACGGGTGGGAACACGTTTTTCAGGTCCTC	7	
P7-I1-SP2[6]	CAAGCAGAAGACGGCATACGAGATATCACGGTGACTGGAGTTCAGACGTGTGCTCTTCCGATC	7	
SP1-P5	AATGATACGGCGACCACCGAGATCTACACTCTTTCCCTACACGACGCTCTTCC	7	
SP2	GTGACTGGAGTTCAGACGTGTGCTCTTCCGATCT	6	
P5	AATGATACGGCGACCACCGAGATCTACACT	7	
P7	CAAGCAGAAGACGGCATACGAGAT	7	
	**MOUSE**		
mAlpha_RC2	GAGACCGAGGATCTTTTAACTGG	3	
mBeta _RC2	GCTTTTGATGGCTCAAACAAGG	3	

mTRAC_Q_F	ACATCCAGAACCCAGAACC	3	
mTRAC_Q_R	GCACATTGATTTGGGAGTCA	3	
mTRAC_Q_Pr	TACCAGTTAAAAGATCCTCGG	3	5’ 6FAM[2]; 3’ BHQ1[3]
mTRBC_Q_F	AGAAATGTGACTCCACCCAA	3	
mTRBC_Q_R	CATTCACCCACCAGCTCAG	3	
mTRBC_Q_Pr	TGCAAACAAACAAAAGGCTACC	3	5’ 6FAM[2]; 3’ BHQ1[3]
mAlpha_RC1	CAGCAGGTTCTGGGTTCTGGATG	7	
mBeta_RC1	GGGTGGAGTCACATTTCTCAGATCC	7	
mSP1-6N-I-6-aRC1	ACACTCTTTCCCTACACGACGCTCTTCCGATCTNNNNNNGCCAAT CAGCAGGTTCTGGGTTCTGGATG	7	
mSP1-6N-I-6-bRC1	ACACTCTTTCCCTACACGACGCTCTTCCGATCTNNNNNNGCCAAT GGGTGGAGTCACATTTCTCAGATCC	7	

1. All oligonucleotides were obtained from Sigma
2. 6-carboxyflouorescein
3. Black Hole Quencher 1
4. N represents a position at which any base can be inserted randomly
5. The underlined bases represent a representative index sequence, and can be substituted by any of the Illumina indices
6. The underlined bases represent a representative index sequence, and can be substituted by any of the Illumina indices

Supplementary Table 3 The correspondence between Decombinator numbering (left column) and standard IMGT nomenclature for human alpha and beta TCR genes

DCR	TCRAV	TCRAJ	TCRBV	TCRBJ
0	TRAV1*01	TRAJ11*01	TRBV1*01	TRBJ1-1*01
1	TRAV10*01	TRAJ12*01	TRBV12-1*01	TRBJ1-2*01
2	TRAV11*01	TRAJ13*01	TRBV12-2*01	TRBJ1-3*01
3	TRAV12-1*01	TRAJ15*01	TRBV13-1*01	TRBJ1-4*01
4	TRAV13-1*01	TRAJ16*01	TRBV13-2*01	TRBJ1-5*01
5	TRAV13-2*01	TRAJ17*01	TRBV13-3*01	TRBJ2-1*01
6	TRAV13-4/DV7*01	TRAJ18*01	TRBV14*01	TRBJ2-2*01
7	TRAV13-5*01	TRAJ2*01	TRBV15*01	TRBJ2-3*01
8	TRAV14-1*01	TRAJ21*01	TRBV16*01	TRBJ2-4*01
9	TRAV14-3*01	TRAJ22*01	TRBV17*01	TRBJ2-5*01
10	TRAV15-1/DV6-1*01	TRAJ23*01	TRBV19*01	TRBJ2-7*01
11	TRAV15-2/DV6-2*01	TRAJ24*01	TRBV2*01	
12	TRAV16*01/DV11*01	TRAJ26*01	TRBV20*01	
13	TRAV17*01	TRAJ27*01	TRBV23*01	
14	TRAV19*01	TRAJ28*01	TRBV24*01	
15	TRAV2*01	TRAJ30*01	TRBV26*01	
16	TRAV21/DV12*01	TRAJ31*01	TRBV29*01	
17	TRAV3-1*01	TRAJ32*01	TRBV3*01	
18	TRAV3-3*01	TRAJ33*01	TRBV30*01	
19	TRAV4-2*01	TRAJ34*01	TRBV31*01	
20	TRAV4-3*01	TRAJ35*01	TRBV4*01	
21	TRAV5-1*01	TRAJ37*01	TRBV5*01	
22	TRAV5-4*01	TRAJ38*01		
23	TRAV6-1*01	TRAJ39*01		
24	TRAV6-2*01	TRAJ40*01		
25	TRAV6-3*01	TRAJ42*01		
26	TRAV6-4*01	TRAJ43*01		
27	TRAV6-5*01	TRAJ45*01		
28	TRAV7-1*01	TRAJ48*01		
29	TRAV7-2*01	TRAJ49*01		
30	TRAV7-3*01	TRAJ5*01		
31	TRAV7-4*01	TRAJ50*01		
32	TRAV7-5*01	TRAJ52*01		
33	TRAV7-6*01	TRAJ53*01		
34	TRAV8-1*01	TRAJ56*01		
35	TRAV8-2*01	TRAJ57*01		
36	TRAV9-1*01	TRAJ58*01		
37		TRAJ6*01		
38		TRAJ9*01		

Supplementary Table 4. Comparison of the analysis of a naïve CD8 TCR repertoire (the same sample illustrated in fig 5a) , analysed using the analysis pipeline described in this paper (results shown in black), and the publicly available analysis tool MIGEC (results shown in parentheses in red).

	**Total TCRs identified**	**Total number of distinct UMI-TCR combinations**	**Total number of distinct TCR**
**alpha**	539403 (595508)	355503 (47917)	31412 (34985)
**beta**	550622 (609045)	65375 (79227)	59001 (63391)
